# Chromatin-dependent regulation of RNA polymerases II and III activity throughout the transcription cycle

**DOI:** 10.1093/nar/gku1349

**Published:** 2014-12-29

**Authors:** Antonio Jordán-Pla, Ishaan Gupta, Lola de Miguel-Jiménez, Lars M. Steinmetz, Sebastián Chávez, Vicent Pelechano, José E. Pérez-Ortín

**Affiliations:** 1 Departamento de Bioquímica y Biología Molecular and ERI Biotecmed, Facultad de Biológicas, Universitat de València, C/Dr. Moliner 50, E46100 Burjassot, Spain; 2 European Molecular Biology Laboratory (EMBL), Genome Biology Unit, Meyerhofstrasse 1, 69117 Heidelberg, Germany; 3 Instituto de Biomedicina de Sevilla (IBiS), Hospital Virgen del Rocío-CSIC-Universidad de Sevilla, and Departamento de Genética, Universidad de Sevilla, 41013 Seville, Spain; 4 Stanford University School of Medicine, Department of Genetics, Stanford, CA 94305, USA; 5 Stanford Genome Technology Center, Stanford University, Palo Alto, CA 94304, USA

## Abstract

The particular behaviour of eukaryotic RNA polymerases along different gene regions and amongst distinct gene functional groups is not totally understood. To cast light onto the alternative active or backtracking states of RNA polymerase II, we have quantitatively mapped active RNA polymerases at a high resolution following a new biotin-based genomic run-on (BioGRO) technique. Compared with conventional profiling with chromatin immunoprecipitation, the analysis of the BioGRO profiles in *Saccharomyces cerevisiae* shows that RNA polymerase II has unique activity profiles at both gene ends, which are highly dependent on positioned nucleosomes. This is the first demonstration of the *in vivo* influence of positioned nucleosomes on transcription elongation. The particular features at the 5′ end and around the polyadenylation site indicate that this polymerase undergoes extensive specific-activity regulation in the initial and final transcription elongation phases. The genes encoding for ribosomal proteins show distinctive features at both ends. BioGRO also provides the first nascentome analysis for RNA polymerase III, which indicates that transcription of tRNA genes is poorly regulated at the individual copy level. The present study provides a novel perspective of the transcription cycle that incorporates inactivation/reactivation as an important aspect of RNA polymerase dynamics.

## INTRODUCTION

Transcription is a highly regulated process that drives gene expression. Therefore, the study of eukaryotic transcription is one of the main topics of molecular biology. For this reason, many *in vivo* and *in vitro* procedures have been developed to study all the steps throughout the transcription cycle (i.e. transcription initiation, elongation and termination) of eukaryotic RNA polymerases (RNA pol; 1,2). A typical organization of strictly positioned nucleosomes is characteristic of the promoters and terminator regions of RNA pol II genes in all studied eukaryotes, including yeast (3,4). Nucleosome presence and positioning influences the initiation, elongation and termination phases in the transcription cycle (3,4). Therefore, eukaryotic RNA polymerases should have evolved to cope with this organization, and they are obviously able to transcribe through nucleosomes. However, nucleosomes act as strong barriers of RNA pol II transcription *in vitro* (reviewed in (1)). *In vivo*, the nucleosomal organization of RNA pol II promoters is variable and depends on the presence or absence of a canonical TATA box (5,6). This variation probably conditions the way RNA pol II initiates transcription (6). Elongation through nucleosomes is dependent on not only their particular features, including positioning, presence of histone variants and histone modifications (7), but also on stimulation by auxiliary factors such as Transcription factor IIS (TFIIS) or others (reviewed in (8)). Current transcription elongation models involve either histone eviction provoked by RNA pol nucleosomal DNA unwrapping or stopping RNA pol before the nucleosome, where it waits for spontaneous nucleosomal DNA unwrapping. Although phage and prokaryotic RNA pol, as well as yeast RNA pol III, can transcribe through nucleosomal DNA by mobilizing histones along templates (9,10), RNA pol II can traverse only the nucleosome under conditions where at least one H2A/H2B dimer is lost (11,12). In this case, histones (either tetramers or hexamers) transfer from downstream to upstream of advancing RNA pol, where the nucleosome rewraps (7). When RNA pol II transcribes through a nucleosomal template, it pauses at certain sites, which are presumably related to the strength or nature of histone DNA contacts (12,13). This pausing leads to RNA pol II backtracking that can be either avoided by Transcription factor IIH (TFIIH) (14) or rescued by TFIIS both *in vivo* (15) and *in vitro* (14).

Most studies into RNA pol elongation through nucleosomes have been conducted *in vitro* (reviewed in (1)) or *in vivo* in specific genes (16). In the advent of genomic methodologies, it is now possible to study both the particular features of every single gene and to determine the real properties of an average gene without having to extrapolate the properties of a particular one to the rest of the genome (reviewed in (17)). In line with this, some high-resolution techniques for studying nascent transcription have been established (18–20). Each technique offers particular features that reveal different aspects of the transcription process (reviewed in (17,21)). For example, chromatin immunoprecipitation (ChIP) detects all RNA pol, regardless of it being active or not, but can differentiate between different RNA pol species or carboxy-terminal (CTD) phosphorylated forms of RNA pol II by using specific antibodies (21). Those techniques that detect nascent RNA (nRNA) measure only elongating RNA pol and enable its high precision mapping (18–20). They are, however, unable to distinguish between active RNA pol II molecules and those that are backtracked, but still retain the bound RNA molecule. The methods that map nRNA may also be biased by the presence of dropped-off RNA polymerase, which might remain bound to its transcribed RNA. Conversely, genomic run-on approaches (GRO; 22,23) detect only active elongating RNA pol I, II and III molecules, and have proven very useful for transcription elongation research (24,25). Therefore, a combination of the results of various independent techniques can prove most useful for determining the proportion of different RNA pol elongating states and, thus, for shedding light on the transcriptional elongation mechanism for different types of genes (17,26). RNA pol I and III have much higher nascent transcription rates (nTRs) than RNA pol II (27–29). RNA pol III transcribes a heterogeneous set of small non-coding RNA genes constituted mainly by tRNA genes. The active transcription of a tRNA gene has been reported to exclude nucleosomes from the gene *in vivo* (28,30). Yet whether the chromatin structure of tRNA genes influences their transcription remains a matter of discussion (3,31).

In this work, we have quantitatively mapped active RNA polymerases at a high resolution following a new biotin-based genomic run-on technique for the model organism *Saccharomyces cerevisiae* based on the use of a modified RNA precursor (biotin-UTP) and tiling microarrays, which enables the specific analysis of active RNA pol molecules for a large set of genes. This novel approach, which we called BioGRO, does not require sample amplification, so it strictly avoids any interference from contaminating mature RNA molecules that can affect the results of previous methods (32). Moreover, lack of an amplification step preserves the quantitative quality of the high-resolution signal by avoiding the intrinsic stochastic noise introduced by amplification protocols. Using BioGRO, we show that RNA pol II elongation activity displays a characteristic pattern along transcribed regions and, by utilizing a mutant strain for chromatin remodeler Isw2, we confirm that nucleosome positioning conditions RNA pol II elongation activity. The comparative analysis of the BioGRO results with those of RNA pol II-ChIP indicates that transcription elongation is influenced strongly by the presence of positioned nucleosomes, especially nucleosome +1, which provokes specific RNA pol II patterns for different gene functional groups. The average gene 3′-end BioGRO profile also displays marked variations in RNA pol II-specific activity, which suggests that backtracking is involved in polyadenylation and transcription termination. We also present the first genome-wide analysis of RNA pol III nascentome, which shows that RNA pol III is poorly regulated at the individual tRNA gene level and that the existence of several gene copies is probably the main cause of differential levels amongst the tRNA species in the cytoplasm.

## MATERIALS AND METHODS

### Yeast strains

All the yeast strains were grown under standard conditions (YPD medium, 30ºC) and derived from the S288c background. BQS252: *Mat* a, *ura3–*52; YOR304W: *Mat a, his3Δ, leu2Δ, ura3Δ, met15Δ, isw2Δ::KanMX4.*

### BioGRO protocol

Briefly, run-on reactions were performed as described elsewhere (33), but with modifications. For each sample, 100-ml cultures were grown to DO_600_ = 0.55. Cells were collected by centrifugation and frozen in liquid nitrogen. Frozen pellets were transferred immediately to −20°C. After at least 3 h, cells were thawed on ice and permeabilized with 10 ml of 0.5% sarkosyl solution. Once permeabilized, cells were split into two aliquots: the first (control sample) was kept on ice, whilst the second (digested sample) was treated with RNase A (Roche). RNase trimming was achieved by incubating cells with 32 μl of RNase A (10 mg/ml) dissolved in 3.2 ml of 0.5% sarkosyl solution under agitation conditions for 10 min at 30°C. In order to remove RNase, cells were washed three times with 50 ml of 0.5% sarkosyl and were then transferred to an eppendorf tube. The cells from the control sample were resuspended in 120 μl of water, whilst the samples treated with RNase were resuspended in 115 μl of water plus 5 μl of RNase OUT to protect the integrity of nRNAs from any residual RNase that might be present. Then 120 μl of 2.5X transcription buffer (50-mM Tris-HCl pH 7.7, 500-mM KCl, 80-mM MgCl_2_), 6 μl 0.1 M DTT (dithiothreitol), 16 μl of NTPs (nucleotides triphosphates) ((adenosine triphosphate) ATP, (cytidine triphosphate) CTP and (guanosine triphosphate) GTP, 10 mM each) and 20.25 μl of 10-mM Biotin-11-UTP (Ambion) were added. The run-on reaction was performed by incubating the mixture at 30°C for 5 min. The reaction was stopped with 1 ml of cold water. Cells were kept on ice for 5 min, harvested by centrifugation and the supernatant (containing unincorporated nucleotides) was removed.

RNA extraction was done using the ‘MasterPure Yeast RNA Purification Kit’ (Epicentre) following the manufacturer's instructions. Once extracted, genomic DNA was removed by digesting with 2 μl of RNase-free DNase I (Roche) for 30 min at 37°C. Purified RNA was resuspended in 32 μl of water and was spectrophotometrically quantified.

### Selective inhibition of RNA pol II

In order to selectively inhibit the enzymatic activity of RNA pol II, the permeabilized cells were incubated 5 min before RNase A degradation with 50 μM of α-amanitin (Sigma). The inhibitor was added again (at the same concentration) as part of the run-on reaction mixture.

### Biotinylated RNA size selection

RNase-degraded labelled nRNAs (<200 nt on average) were separated from the larger, unlabelled molecules using the miRNA NucleoSpin microRNAs isolation kit (Macherey-Nagel) following the instructions in Section Purification of siRNA from DICER and large dsRNA reactions: purification of siRNA from DICER and large dsRNA reactions. Small RNAs were eluted from the column in 30 μl of water and were spectrophotometrically quantified (see Supplementary Figure S1b).

### Radioactive genomic run-on

The radioactive run-on was performed as described in García-Martínez *et al.* (33). RNase degradation was done as described above.

### Northern blot from agarose gels

After agarose gel electrophoresis, performed under native or denaturing conditions, RNA was transferred to positively charged nylon membranes by northern blotting, as described in Sambrook and Russell (34). Membranes were cross-linked with 50 mJ of ultraviolet radiation with a bench-top Stratagene Crosslinker (BioRad).

Once cross-linked, the biotinylated RNA-containing membranes were treated as follows: the membrane was blocked with blocking buffer (10% sodium dodecyl sulphate, 125-mM NaCl, 7-mM monosodium phosphate, 9-mM disodium phosphate) for 20 min at room temperature, incubated with Streptavidin-HRP (Thermo-scientific, 1:4000 in blocking buffer) for 10 min at room temperature (RT), washed twice with blocking buffer and twice with wash buffer (0.1-M Tris-HCl pH 9.5, 0.1-M NaCl, 10-mM MgCl_2_). An ECL chemiluminescent system (GE Healthcare) was used to develop the signal, which then was acquired with the ImageQuant LAS 4000 system (GE Healthcare).

For the radioactive samples, membranes were plastic-sealed, exposed to phosphor imaging plates (IP) screens and scanned with a Phosphorimager system (Fuji).

### Northern blot from polyacrylamide gel electrophoresis gels

For the size separation of biotinylated RNAs, 8% polyacrylamide gels (TBE-urea 7 M) were used. Separated RNAs were transferred to nylon membranes by the ‘Wet’ method with the MiniProtean Tetra System (BioRad) in 0.5X TBE for 90 min at 100 V in a cold room. The membrane was then dried between paper sheets, cross-linked with UV light and processed as described in the previous section.

### Tiling array hybridization

From each BioGRO sample, at least two biological replicates (see Supplementary Figure S3a) were hybridized to Custom Tiling Array (PN 520055, Affymetrix, Santa Clara, CA, USA) (35). Then 5 μg of BioGRO nRNAs were hybridized directly on the chip following the GeneChip® Whole Transcript (WT) Sense Target Labelling Assay Manual, but skipping the cDNA and amplification steps. To increase signal intensities, an additional pass of staining and chip re-scanning was performed.

### RNA pol II ChIP

For the Rpb3 ChIP-on-chip experiments, ChIP was performed as previously described (36). Immunoprecipitation (IP samples) was performed with magnetic beads (Dynal) using the antibody Rpb3 (ab81859, Abcam) and, after sonication and crosslinking reversal, the obtained fragments (300 bp approximately) of enriched DNA were amplified non-specifically and labelled following the Affymetrix ChIP Assay Protocol (Affymetrix, P/N 702238). Genomic DNA controls (‘Input’ samples) were processed in parallel. After polymerase chain reaction (PCR) amplification with dUTP, samples were purified with the Qiagen QIAquick PCR Purification Kit (50) (Qiagen). DNA was spectrophotometrically quantified in a NanoDrop ND-1000 Spectrophotometer (Thermo Scientific) and 0.5 μg of DNA per sample were hybridized to the same Custom arrays as the BioGRO samples.

### cDNA labelling

cDNA hybridizations for ‘total RNA’ samples were taken from (37).

### Nucleosome mapping

Translational positioning of nucleosomes was mapped genome wide by digesting formaldehyde-fixed chromatin with micrococcal nuclease (MNase). Mononucleosomal DNA from each sample, used to create sequencing libraries, was subjected to 36-nucleotide single-read sequencing in a Solexa Genome Analyzer IIx. Nucleosome maps were generated with the DANPOS comprehensive bioinformatics pipeline (38). MNase-digested naked DNA controls were performed in order to improve map resolution. A more detailed description of this method will be published elsewhere (G. Gutierrez *et al.*, in preparation).

### Tiling array bioinformatics analysis

The raw .CEL images were processed by the Tiling Analysis Software (TAS, Affymetrix) with the signal detection parameters set by default. To visually inspect the hybridization signals in relation to the annotations from the *S. cerevisiae* reference genomic map, the Integrated Genome Browser (http://bioviz.org/igb.html) software was used. The ‘TilingArray’ Bioconductor (http://www.bioconductor.org/packages/2.11/bioc/html/tilingArray.html) and custom R scripting packages were used for the metagene analysis, scatterplot generation and k-means clustering. The ‘simple tiling array analysis of Affymetrix ChIP-chip data’ (STARR, 39) was also used to analyse the RNA pol II ChIP-chip data.

All the BioGRO samples were normalized against genomic DNA (YJM789 strain) hybridized into the same custom Affymetrix arrays (as in 40). The Array Express accession number is E-TABM-470.

The total RNA hybridization used for plotting and comparing against the BioGRO, RNA pol II and nucleosome data was downloaded from (37). The Array Express accession number is E-TABM-590.

## RESULTS

### Transcriptional run-on across the genome of *S. cerevisiae* at a high resolution

We previously developed a GRO protocol that uses ^33^P-UTP to label nRNA, which is hybridized to nylon macroarrays (22). These macroarrays contain PCR fragments that expand the whole open reading frame (ORF) sequence for most *S. cerevisiae* genes (41). The GRO method is a fast, simple and very efficient way of determining the average elongating RNA pol II density for the whole set of yeast genes in many experimental circumstances using a small amount of cells (17). These average densities are converted into nTRs by assuming constant elongation speed (42). However, the distribution of the RNA polymerases inside the ORF cannot be analysed with GRO. Variants of GRO have been published later by other laboratories that work on yeast or higher eukaryotes (23,32,43,44). All these methods require an amplification step before analysing purified nRNA. Purification of very rare nRNA requires having to label it with a precursor, such as BrUTP or Biotin-UTP. Given the small proportion of nRNA in the cell (between 0.05 and 0.34% in yeast, according to (18) and (32)), contamination with mature RNA is a major concern. The unnoticed presence of such a contaminant, and the requirement of amplification steps, may obscure the conclusions drawn from all current methods that measure nascent transcription.

**Figure 1.**
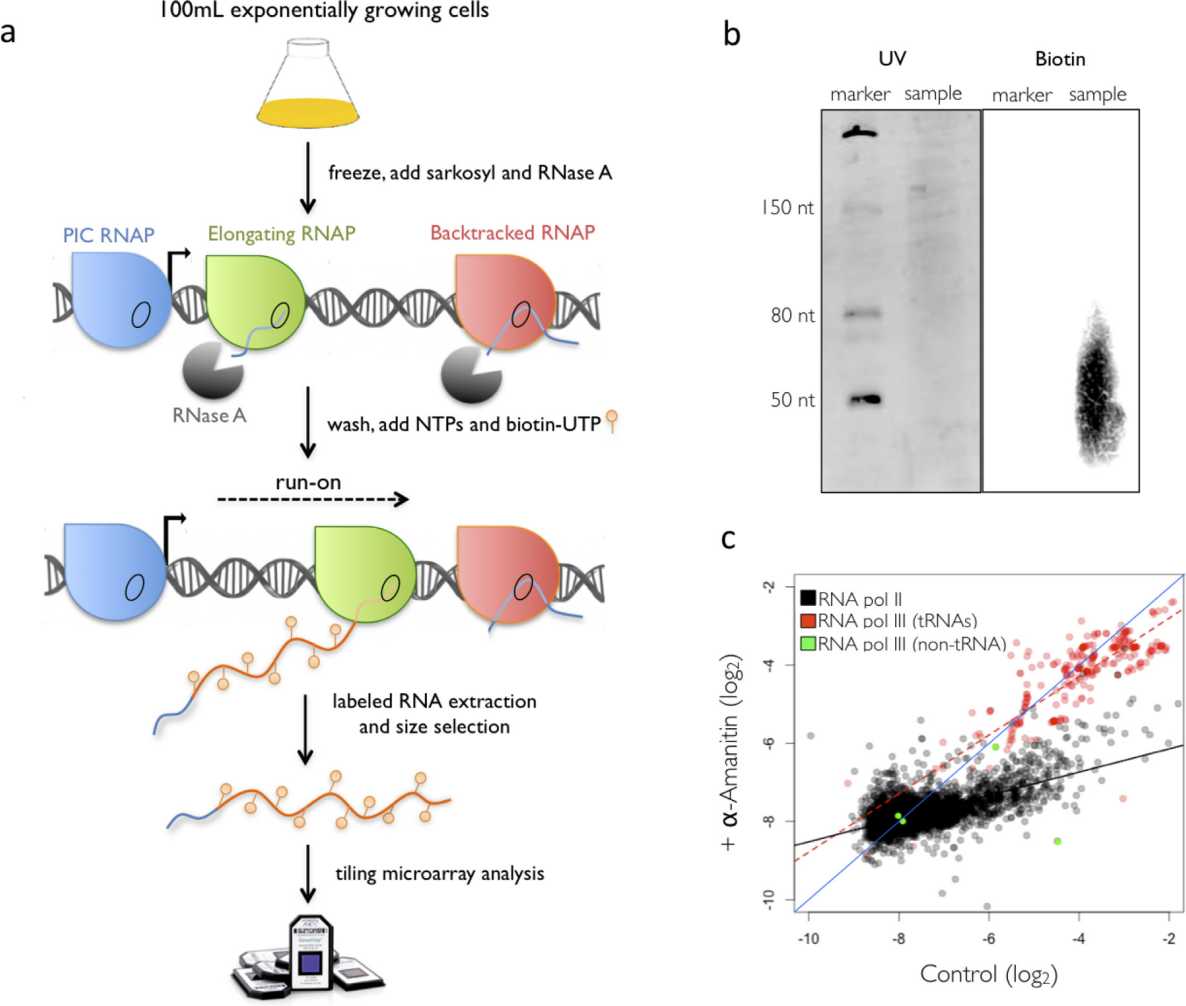
Outline of the BioGRO method. (**a**) The colours of RNA polymerases (RNAP) represent different transcriptional states. Only active elongating RNA pol II (green) is able to run-on. (**b**) Analysis of the size of the run-on elongation with Biotin-UTP. Biotinylated RNAs appear as a diffuse luminescent stain signal centred at about 50 nucleotides (nt). (**c**) BioGRO after a selective inhibition of RNA pol II with α-amanitin. The graph shows the comparison made between BioGRO signals in the presence or absence (control) of α-amanitin. In black, the RNA pol II genes and their trend line. The tRNA genes and their trend line are depicted in red. Green dots mark the RNA pol III transcripts that do not belong to the tRNA type.

We modified the existing Genomic-run on (GRO) protocol to generate biotin-labelled nRNAs, which we then used to directly hybridize Affymetrix tiling arrays. We called it Biotin-GRO or BioGRO (Figure 1a). In order to reduce the amount of contaminant RNA and improve hybridization, we treated sarkosyl-permeabilized cells with RNase A. In addition to eliminating most of the mature RNA present in the cell, RNase A treatment also trims the 5′ tail of nRNA to confer an RNA polymerase footprint of ∼25 nucleotides (nt) without affecting run-on efficiency (45). Under our experimental conditions, the average labelled nRNA size was 50 nt (Figure 1b). This small fragment average enabled more precise mapping and was, thus, indicative that run-on extends for ∼25 nt on average. This length is shorter than that observed using radioactive nucleotides (García-Martínez J. and Pérez-Ortín J.E., unpublished; 46), but is coherent with the well-known difficulty of RNA polymerases of using Biotin-nucleotides (44), which suggests there are only a few biotin-nucleotides in each labelled nRNA (see Supplementary Figure S1a). We compared our results with those of McKinlay *et al.* (32) and found that our method is less biased towards 5′, likely due to the RNase A 5′-trimming. Our procedure, thus, provides shorter nRNAs and increases method resolution (Supplementary Figure S2). To account for RNA pol average downstream displacement during run-on, we corrected all the BioGRO maps by a 25 nt upstream 5′ shift. We investigated whether there was any significant bias due to the different Uracil content of each fragment or not, and we did not find any (Supplementary Figure S3b). We also corrected any possible hybridization differences by normalizing raw BioGRO signals by random primed genomic DNA (see the Materials and Methods section).

To confirm that the observed fluorescence signal was due to nRNA, we compared the BioGRO results with those of conventional cDNA hybridization. Supplementary Figure S4 shows that the BioGRO signal was present at both introns and exons with a similar intensity, whereas the cDNA signal was absent in introns, as expected. BioGRO was also able to detect antisense (AS) transcription (37). It is interesting to note that canonical genes with known AS transcription (47) showed a poorer sense signal on average than the genes without AS (Supplementary Figure S5).

The average BioGRO signal for 809 yeast ORF-containing genes, rRNA gene and all the RNA pol III genes in a wild-type strain growing in the log phase was at least >6 times above the background. For some analyses, we focused on this set of confident genes to improve the profiles quality. The BioGRO high-resolution data can also be used to quantify nTR as in conventional radioactive GRO experiments. We averaged signal intensity within the ORF to obtain a single nTR value. The plots of those nTR against the transcriptomics data from other sources (Supplementary Figure S6) showed that our per gene signal best correlated with previous GRO results (27) whilst, as expected, plotting against either the mRNA amount data (RA; 48) synthesis rate for mature mRNA data (DTA; 49) or the RNA pol II ChIP data (this paper) gave poorer correlations.

To further prove that BioGRO measures nRNA, we treated cells with α-amanitin, a peptide which, at low concentrations, specifically inhibits RNA pol II (50). The cells treated with α-amanitin showed markedly diminished signals for RNA pol II genes, but did not affect the signals for RNA pol III (Figure 1c). The sudden relative reduction in the BioGRO signal on protein-coding genes proves that it came from nRNA and was not caused by mature mRNAs. Thus, BioGRO is an efficient method to detect nRNA and does not present significant contamination from mature RNA.

### A high-resolution profile of RNA pol II elongating activity for protein-coding genes

**Figure 2.**
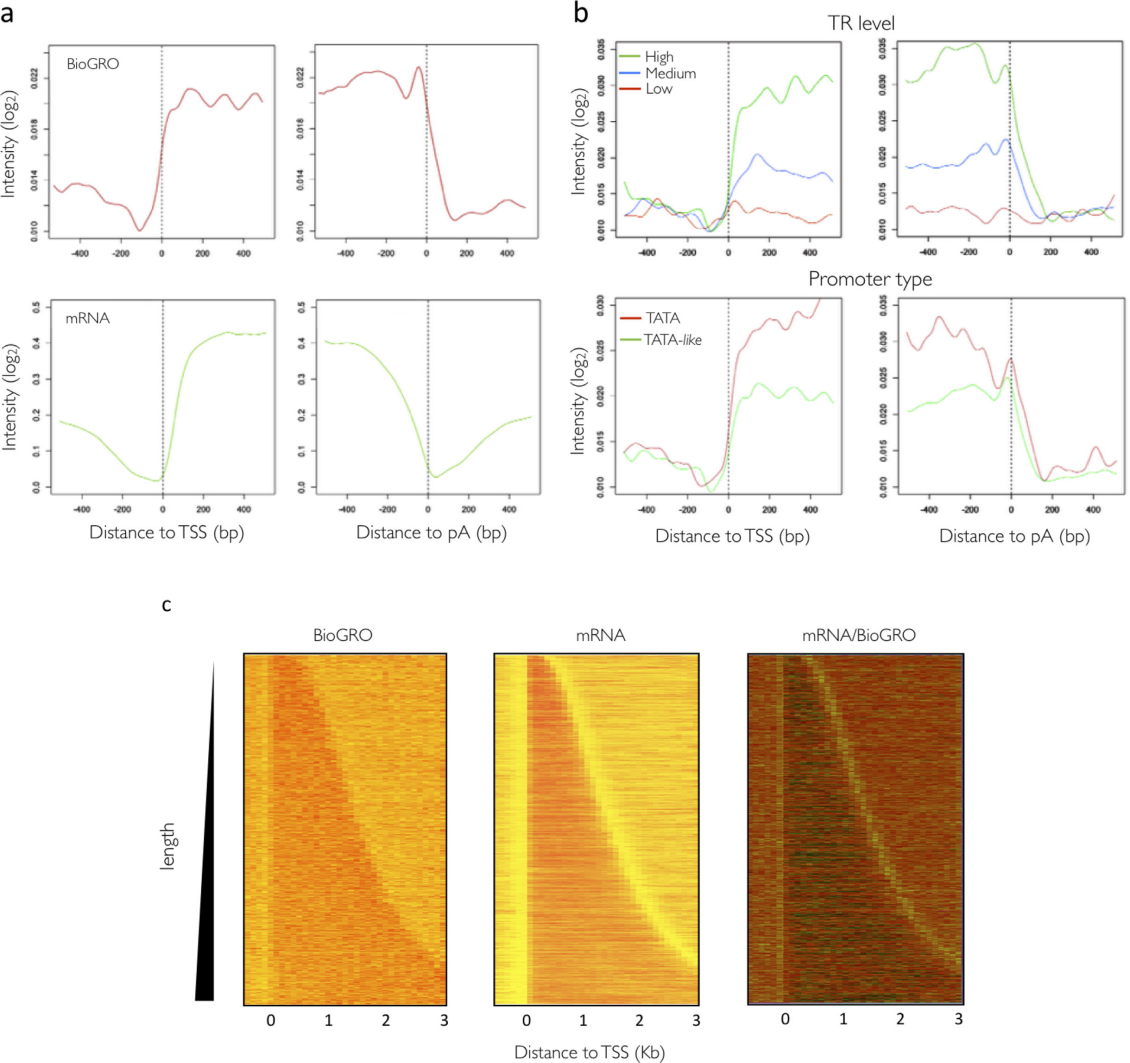
BioGRO average profiles for the 5′ and 3′ ends of yeast genes. (**a**) Comparison of average nascent transcript profiles (top) and mature mRNA (bottom; 37) on a log_2_ scale of arbitrary intensity units. Vertical dotted lines mark the transcription start sites (TSS) and polyadenylation sites (pA). (**b**) BioGRO profiles for the different groups of genes classified according to their transcription rates (TR, top) or to promoter type (bottom). (**c**) Heat map comparison made between the data of the nascent and mature mRNAs for the individual genes sorted by size. The lighter areas in the right panel (mRNA/BioGRO) reflect the ratio of the mRNA signal versus nRNA.

One advantage of BioGRO over previously published methods for nRNA detection is that RNA pol activity itself is utilized to map its position. Since the use of tiling arrays allows BioGRO to determine the transcriptional activity of RNA pol II at a high resolution, we decided to focus on those regions where transcription regulation was expected, such as the transcription start site (TSS), the polyadenylation site (pA) and intron boundaries. In the 5′ region, the BioGRO average profile clearly differed from the mRNA profile (Figure 2a, left panels). The signal started upstream of mRNA. The BioGRO profile around the TSS also showed that the ‘poised’ RNA pol II molecules in yeast did not generally accumulate, contrarily to what has been detected in animals (see (21)). This is probably due to the advanced position of nucleosome +1 in yeast (6). The average BioGRO profile around the pA was also distinctive from mRNA (Figure 2a, right panels).

We wondered if all kinds of genes displayed similar 5′ and 3′ BioGRO profiles. To test this, we separated the data set into different gene groups according to features such as nTR or presence of TATA box (Figure 2b). First, when dividing genes according to their nTR level, we saw that the BioGRO signal increased from 5′ to 3′, and that it was accompanied by a wavy pattern in the 5′ region (see below). These features were better seen in the high nTR genes than in the medium or low nTR genes. One interesting point was that a class of high nTR genes, ribosomal protein (RP)-coding genes, displayed a distinctive profile in both 5′ and 3′, with sharper peaks and an abrupt drop in signal from the TSS onwards (Supplementary Figure S7a), as previously reported for a small group of them (51). It was also remarkable that the TATA-containing and TATA-like genes (6) showed slightly different profiles in both the 5′ and 3′ ends (Figure 2b). It is noteworthy that the TATA genes were more expressed than the TATA-like genes on average, as recently reported by Eser *et al.* (52) in a yeast cell cycle transcription study.

In order to better analyse the results on both gene ends, we plotted gene length-ordered heat maps for the BioGRO, mRNA and BioGRO/mRNA data. Figure 2c shows how the BioGRO signals extended about 70 bp upstream of the mRNA signal at the TSS and 150 bp downstream of the mRNA signal in the pA region. The upstream signal can be attributed to cryptic sense transcription (53), to which BioGRO was much more sensitive than conventional RNA analyses. The comparison made with chromatin-associated nRNA (19) confirms the biological origin of this extension (Supplementary Figure S8). The differential presence of RNA pol II beyond the pA site possibly indicates the actual transcription termination site (TTS) positions, as discussed later.

Finally, the intron-containing genes analysis (Supplementary Figure S9) showed a relative accumulation of elongating RNA pol II in the 3′ end of the transcribed region. This result is compatible with the observation made by Carrillo-Oesterreich *et al.* (19) who, based on a different nRNA mapping procedure, proposed a slowdown of RNA pol II molecules at the final exon to allow for co-transcriptional splicing (25,54,55).

### Nucleosome positioning in 5′ shapes RNA pol II dynamics

**Figure 3.**
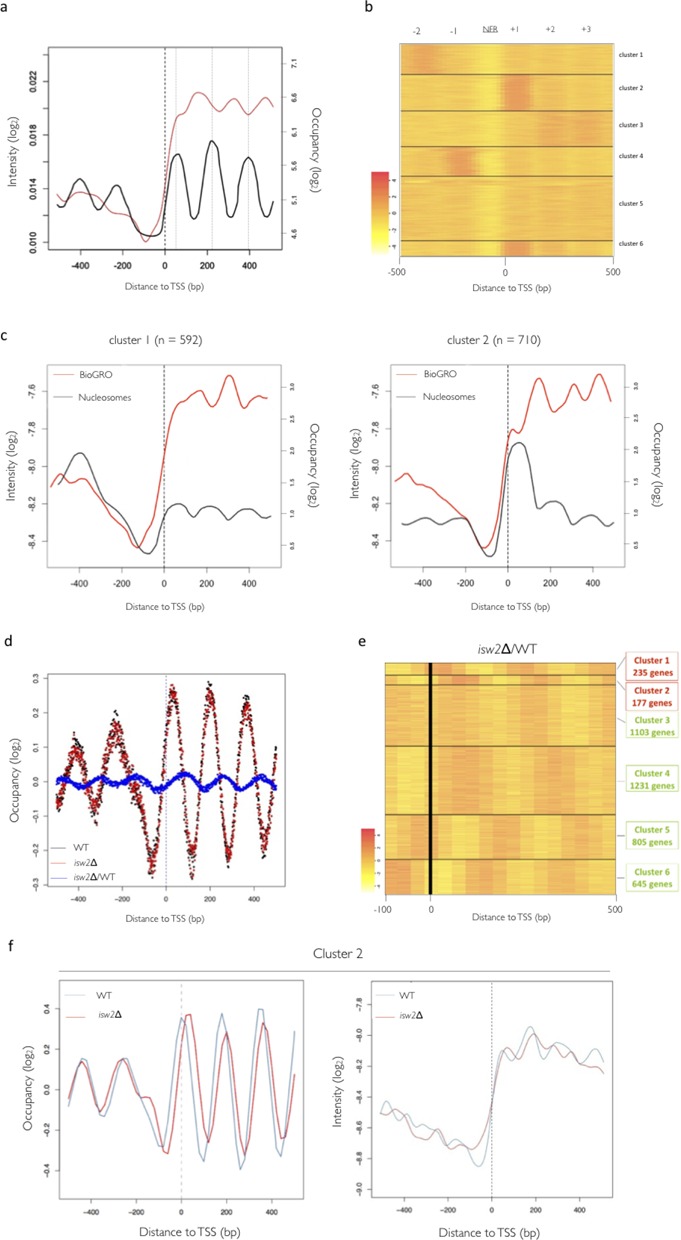
In-depth analyses of BioGRO dependence on 5′ end nucleosomal organization. Comparison of the average nRNA profiling data from total RNA pol II and nucleosome positioning. (**a**) BioGRO average profile (red line, on the log_2_ scale of arbitrary intensity units) compared to the average nucleosome positioning (black line, on the log_2_ scale of occupancy levels) obtained from (G. Gutiérrez, S. Chávez *et al.*, in preparation). (**b**) Re-analysis of the data on the global positioning of nucleosomes from (98): heat map profile similarity grouping by k-means. Similar results were obtained when using other nucleosome positioning data sets (not shown). (**c**) Relationship between chromatin structure and RNA pol II activity. The graphs show overlapped profiles of the genes in clusters #1 and #2 derived from the cluster analysis by k-means and their average BioGRO profiles. (**d**) Positioning of nucleosomes on the genomic scale aligned by the TSS and the resulting profile of the ratio between the *isw2*/WT data (blue dotted trace). (**e**) Heat map of the gene clustering resulting from the displacement of the nucleosomes between the WT and mutant strain. Colour intensity in arbitrary log_2_ units. (**f**) Analysis of nucleosome re-positioning and the BioGRO profile in the *isw2* mutant. The figure on the left shows the profile of the nucleosomes aligned by the TSS to genes cluster #2 obtained by k-means. The right-hand graph is the corresponding 5′-end BioGRO profile.

BioGRO profiles showed a regularly spaced peak-and-valley shape from the 5′ start towards the gene body (Figure 3a, red line). These peaks reached their maximum height approximately every 165 bp, and alternated almost perfectly with the average +1, +2 and +3 positioned nucleosomes (Figure 3a, black line), which suggests that nucleosomes may influence RNA pol II elongation activity. To dissect the crosstalk between transcription elongation and nucleosome positioning, we decided to further analyse the BioGRO profile around the 5′ end. The peak before nucleosome +1 is presumably blurred by the fact that it partially overlaps the TSS in yeast (6,18), but a shoulder can be hinted at by the dyad of the nucleosome +1 peak.

In order to check if the BioGRO profile reflected interplay between RNA pol and nucleosomes, we decided to separate genes by their 5′-end nucleosomal profiles. We used the improved MNase genome mapping data of yeast nucleosomes (98) to divide genes into six clusters with distinct nucleosomal positioning profiles, as shown in Figure 3b. Clusters 1 and 2 represent the most extreme alternatives for nucleosome +1 positioning. Figure 3c shows that the BioGRO profile for the genes in both clusters differed for the region up to 100 bp downstream of the TSS. The average BioGRO signal in the genes with a strongly +1 positioned nucleosome (Cluster #2) was reduced if compared to that of those with a slight positioning for it (Cluster #1). This supports the hypothesis that positioned nucleosomes influence the active RNA pol II density profile.

As a complementary approach to the same question, we decided to test whether specific variations in nucleosome positioning were reflected in the BioGRO profile. To do this, we used an *isw2* mutant strain. Isw2 is a chromatin remodeler that spatially arranges nucleosomes at both ends of the transcribed region in a subset of yeast genes (56,57). If nucleosome positioning is the cause of variation in RNA pol II elongation activity, a change in nucleosome positioning is expected to be accompanied by a displacement in the elongating RNA polymerases pattern. Using previously published data (56), we clustered yeast genes according to their variation in nucleosome positioning upon Isw2 deletion (Figure 3d and e). Cluster 2, composed of 177 genes, displayed the most marked displacement for the first four positioned nucleosomes (Figure 3f, left) and, as predicted, a 10–20-bp displacement of the *isw2* BioGRO peaks took place (Figure 3f, right, red line) compared with the wild-type (WT) ones (Figure 3f, right, blue line), of a similar magnitude to that observed for the positioned nucleosomes (Figure 3f, left).

Taken together, all these results strongly suggest that the wavy BioGRO profile represents the influence of the positioned nucleosomes along the 5′ end of the transcribed region on RNA pol II dynamics.

### BioGRO-RNA pol II ChIP comparison reveals the singularity of nucleosome +1 in backtracking terms

**Figure 4.**
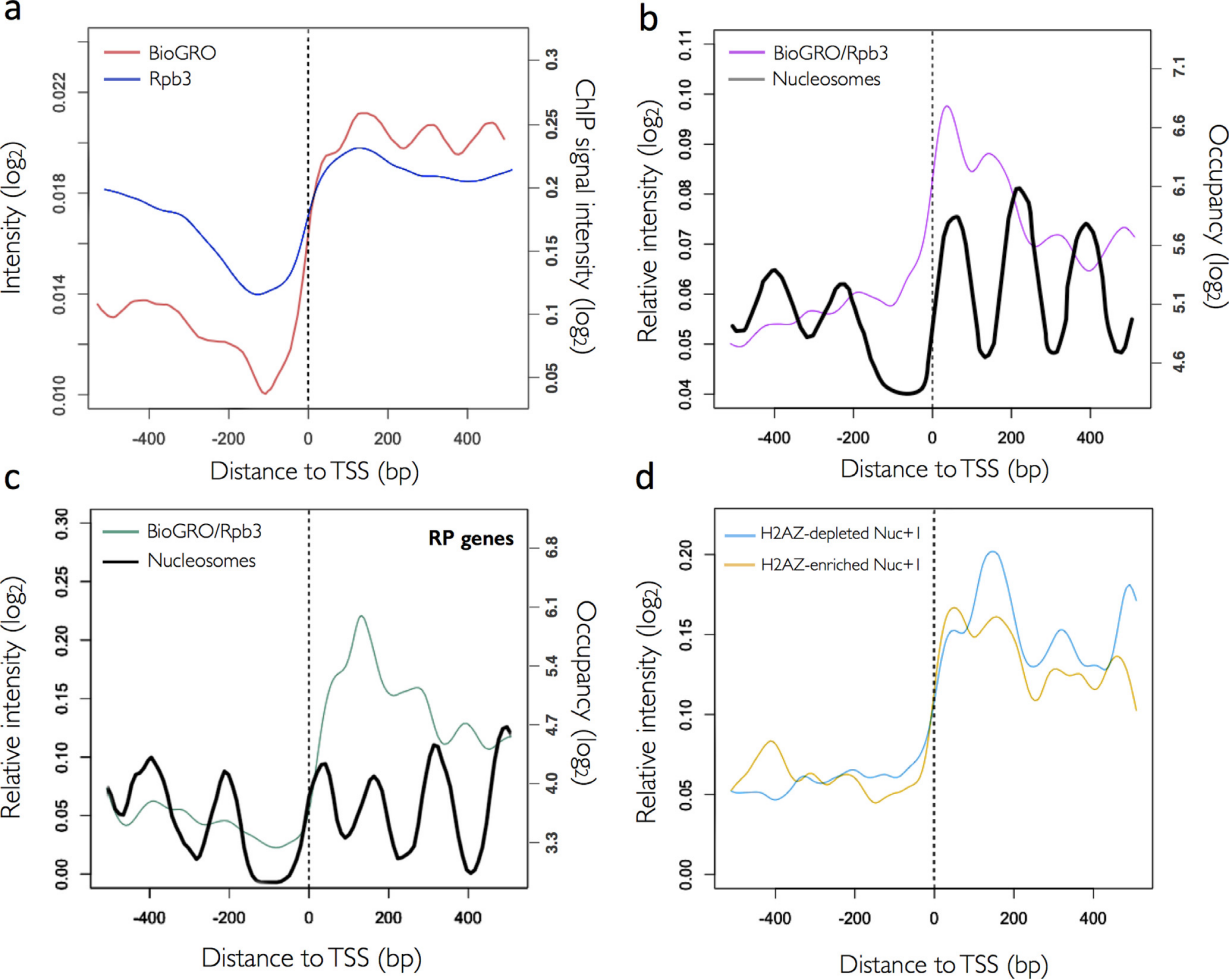
Comparison of BioGRO and RNA pol II-ChIP profiles. (**a**) BioGRO (red line) and RNA pol II-immunoprecipitated using an anti-Rpb3 antibody (blue line; on a log_2_ scale of arbitrary intensity units). (**b**) Ratio for the BioGRO and Rpb3 profiles from panel (a) (purple line) compared to the nucleosome profile (black line). (**c**) Same as in (b), but for ribosomal protein (RP) genes. (**d**) BioGRO/Rpb3 profiles for the 809 most highly transcribed genes enriched (top third in the H2AZ/nucleosome ratio; blue line) or depleted (bottom third in the H2AZ/nucleosome ratio; gold line) in the H2AZ histone variant (99). Other symbols are the same as in Figure 3.

Since the BioGRO signal originates exclusively from transcriptionally active RNA pol, the characteristic profile in the 5′ region may reflect backtracking-mediated RNA pol II inactivation induced by nucleosomes. The presence of the RNA pol II molecules that are incompetent for run-on might be also caused by an alternative change in the elongating RNA pol II molecule, and without requiring backtracking. Although formally possible, this hypothetical conformation has not been described to our knowledge. Nucleosomes have been clearly connected to RNA pol II backtracking *in vitro* (12). Therefore, we consider that the comparison of Bio-GRO and Rpb3-ChIP profiles provides valuable information on the relative proportion of backtracked RNA polymerases (see (58) for a review). As previously pointed out, the ‘antinucleosomal’ BioGRO profile has not been seen in either our RNA pol II (Anti-Rpb3) ChIP data (Figure 4a, blue line) or previously published RNA pol II ChIP data (6,59).

In order to facilitate the comparison between BioGRO and RNA pol II ChIP, we calculated the BioGRO/Rpb3-ChIP ratios. The higher the ratio, the lower the proportion of backtracked RNA pol II became. It is, therefore, a measurement of its specific elongation activity. Figure 4b shows the average BioGRO/Rpb3-ChIP ratio along the 5′ region. We found a wavy profile that was maximal at the beginning, but continuously decreased as it moved away from the TSS. The profile shows that RNA pol II-specific activity peaked in the linker regions amongst nucleosomes +1, +2, +3 and +4 (Figure 4b) and that an additional peak centred at nucleosome +1 appeared at the very beginning. In line with this, nucleosome +1 gave the highest BioGRO/Rpb3-ChIP ratio, which indicates very strong specific activity and, according to our interpretation, a very low backtracking rate in this nucleosome.

We previously described that RP genes were characterized by excess backtracked RNA pol II molecules (24,26). This was confirmed by the biased position of this group of genes when the overall BioGRO data were plotted against the RNA pol II ChIP data (Supplementary Figure S6d). A comparison of the 5′ BioGRO and RNA pol II ChIP profiles was also informative. Whereas the BioGRO signal diminished towards the gene body, the ChIP signal increased (Supplementary Figure S7a). The canonical TATA genes exhibited the opposite pattern (increasing Bio-GRO and a decreasing ChIP signal), whereas the TATA-like genes displayed parallel profiles for the two signals (Supplementary Figure S7c). It is noteworthy that the BioGRO/ChIP ratio of the RP genes showed a very characteristic profile (Figure 4c). A wavy pattern of peaks was also observed, but their positions shifted upstream, which is in agreement with the shorter nucleosome spacing of RP genes (5). This coordinated shift of BioGRO/ChIP peaks and nucleosomes once again confirms the influence of nucleosomes on RNA pol II activity. The absolute BioGRO/ChIP maximum of the RP genes did not map in nucleosome +1, but in their +1/+2 linker region (Figure 4c), indicating that all the positioned nucleosomes in RP genes, including +1, favour RNA pol II backtracking (24).

The above results suggest that nucleosome +1 is a regulatory point for RNA pol II backtracking. Nucleosome +1 is known to be enriched in histone variant H2AZ (60). Interestingly, RP genes are poorly occupied by H2AZ (61). H2AZ nucleosomes are prone to being displaced during transcription elongation (62,63). Therefore, we classified highly expressed yeast genes according to their H2AZ-specific richness (H2AZ/H3 ratio) and represented the BioGRO/RNA pol II profile of the richest and poorest thirds (Figure 4d). These two groups of genes, which were highly transcribed, but differed in their +1 H2AZ relative content, exhibited different BioGRO/RNA pol II profiles with higher ratios in nucleosome +1 for the H2AZ-enriched genes (99). We conclude that nucleosome +1 regulates RNA pol II backtracking, likely by an H2AZ-dependent mechanism.

### Detailed analysis of the 3′ region: implications for transcription termination

**Figure 5.**
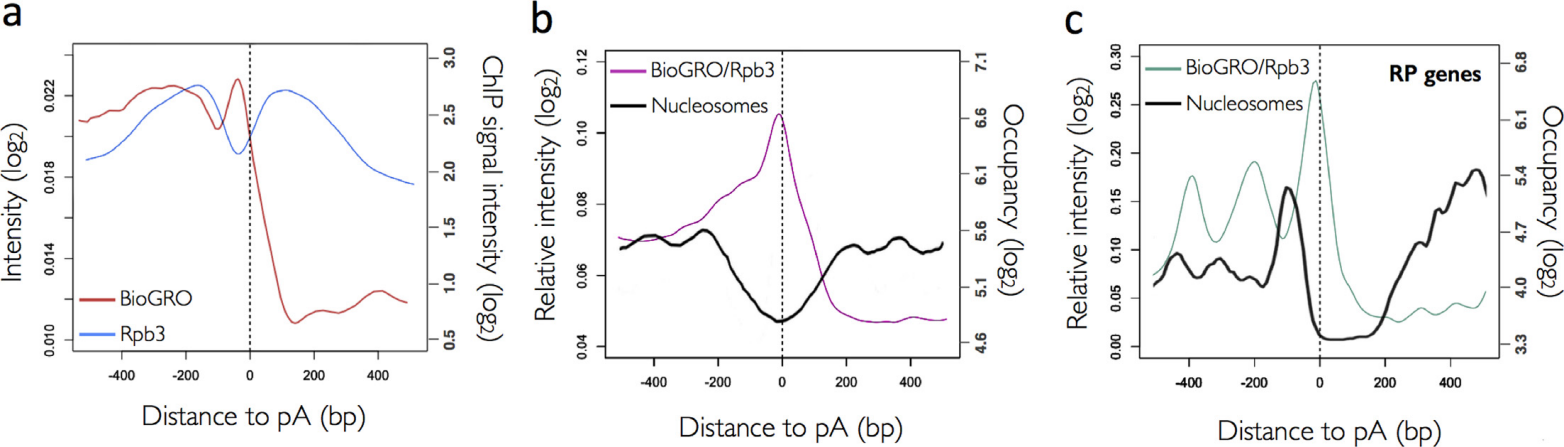
The BioGRO profile around the poly(A) site. (**a**) Average BioGRO (red line) and Rpb3 (blue line) profiles. (**b**) Average profile resulting from the BioGRO/Rpb3-ChIP ratio in the 3′ end of genes (purple line) compared to the nucleosome positioning average profile (black line). (**c**) Same as in panel (b), but for ribosomal protein (RP) genes. Other symbols are the same as in Figures 3 and 4.

After dissecting the dynamic changes associated with transcription initiation and early elongation steps, we decided to investigate regulation at the polyadenylation and termination stages of the transcription cycle. The BioGRO profile in the 3′ region (Figure 5a, red line) showed two distinctive peaks: the upstream one was wider and centred at about −250 from the pA site; the second one was sharper and placed some 25 nucleotides before the pA site. The first peak coincided approximately with the upstream peak for the total RNA pol II shown by ChIP (Figure 5a, blue line). The second peak, however, was placed precisely at the valley in between the two RNA pol II peaks detected in the BioGRO profiles and the Polymerase ChIP profiles (59).

**Figure 6.**
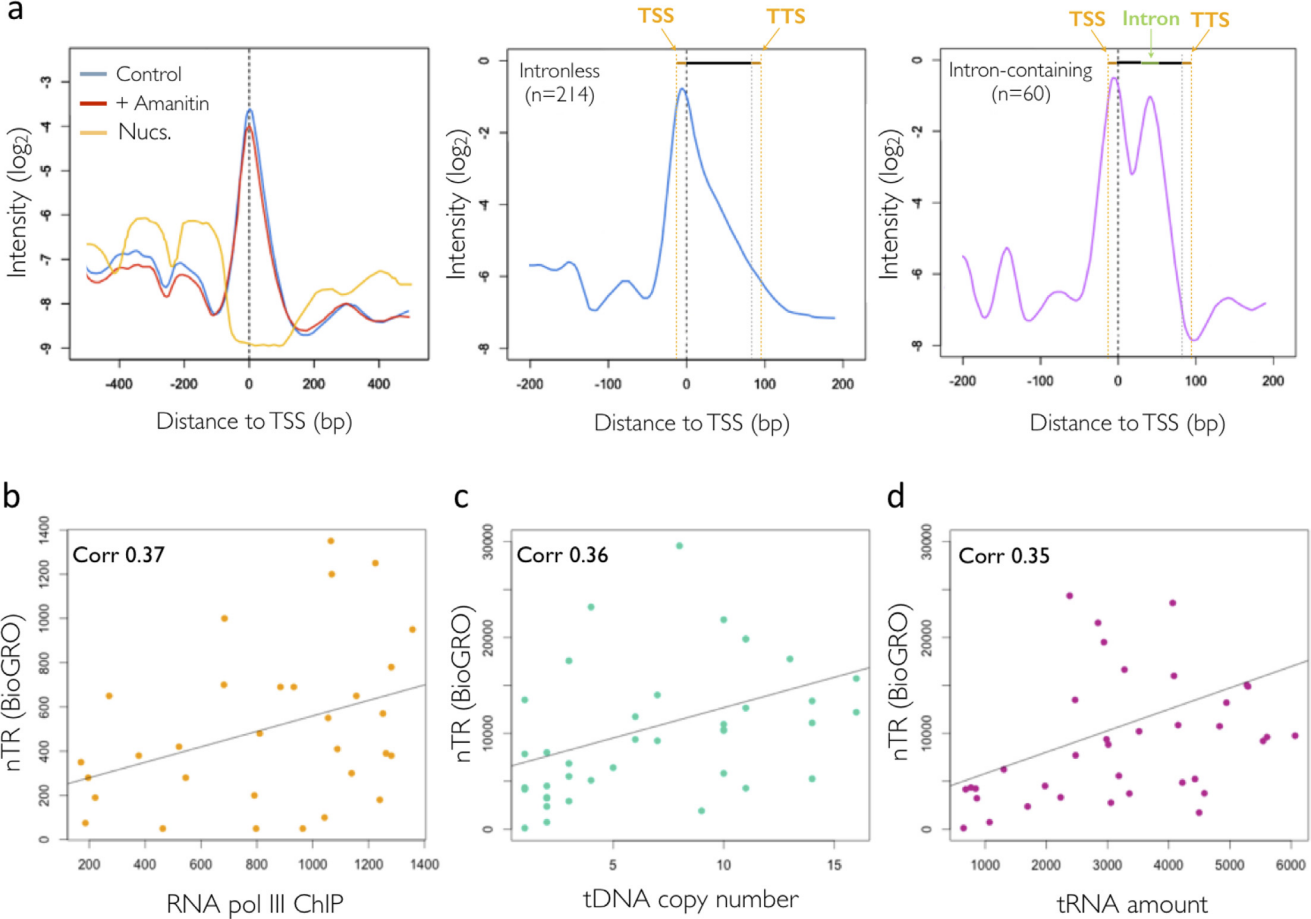
RNA pol III nascentome. In the upper part: (**a**) left panel: analysis of a metagene 5′ assay after the selective inhibition of RNA pol II with α-amanitin. Central panel: metagene tRNAs genes without intron. Right panel: metagene genes of tRNAs with introns. The entries at the top of both figures refer to the pre-tRNA structure: extensions 5′ and 3′ in orange, green introns. In the lower part, the correlations between: (**b**) the RNA pol III ChIP data from Kumar and Bhargava (31) and the BioGRO data; (**c**) the average nTR measured by BioGRO tRNAs of each family and the number of copies of the genes that make each family; (**d**) between BioGRO nTR and the amount of tRNAs for each family, taken from Tuller *et al.* (94).

The extension observed in the BioGRO signal after the mapped poly(A) sites (Figure 2c) was coherent with current termination models (64,65), in which RNA pol II extended several hundred nucleotides after the pA site. To study the variations in the specific RNA pol II elongation activity in the termination region, we represented the BioGRO/RNA pol II ChIP ratio (Figure 5b). This ratio showed a distinctive profile with a peak placed a few nucleotides before the pA site, as well as a continuous decrease after reaching the background level at around 150 bp downstream. The BioGRO/RNA pol II value at the pA site coincided almost exactly with the ratio value on nucleosome +1 in the 5′ region (Figure 4b). In this case, the peak in the RNA pol II-specific activity mapped in the nucleosome-depleted region was localized downstream of the −1-positioned nucleosome of the pA area (Figure 5b). This was observed even more clearly in RP genes. This gene category exhibited a wavy BioGRO/RNA pol II profile in the 3′ end of the gene body, which alternated with the positioned −3, −2 and −1 nucleosomes (Figure 5c). The BioGRO/RNA pol II ratio maximum in RP genes was also mapped in the nucleosome-depleted region downstream of nucleosome −1 (Figure 5c). This suggests that the RNA pol II at the pA site no longer tended to backtrack at this precise site, which it acquired immediately after transcribing nucleosome +1. The functional meaning of this maximum in the proportion of active RNA pol II at the pA site is not altogether clear to us, but might contribute to the polyadenylation machinery recognizing the pA sequence, to transcription termination, or even to both (see the Discussion section). In any case, after the pA site, the BioGRO/RNA pol II ratio dropped immediately in parallel with the total RNA pol II accumulation detected by ChIP. This broad RNA pol II ChIP peak (Figure 5a; (6,59)) can be caused, in part, by the lower resolution of this technique, but it certainly reflects the accumulation of inactive RNA pol II after mRNA cleavage and polyadenylation.

### RNA pol III nascentome

BioGRO also provided us with information about other nuclear RNA polymerases. Figure 1c shows that the signal from tRNA genes was preserved after α-amanitin poisoning, whereas it strongly decreased in most of (or all) the known RNA pol II genes. This confirms that RNA pol II is responsible for most of the run-on signal along the yeast genome, whereas those regions known to be transcribed by RNA pol III have an α-amanitin-resistant run-on signal, as expected.

The metagene plot of tRNA genes (Figure 6a, left) showed a strong α-amanitin resistant peak (>50 times more intense than the average RNA pol II gene) placed in between the positioned nucleosomes (66). The BioGRO peak within the tRNA genes was placed at the TSS. In fact this specific position coincided with that observed (but at a lower resolution) for RNA pol III by ChIP ((31); Supplementary Figure S4), which indicates either a higher elongation speed or the drop-off index from the initiation of transcription towards the 3′ end of these genes. The separated analysis of intron-containing and intronless tRNA genes presented an interesting difference (Figure 6a, centre and right). There was a peak in the intron region which was not visible in intronless genes. Given that pre-tRNA splicing in yeast is not co-transcriptional, but cytoplasmatic (67), the increased presence of elongating polymerases in the intron region suggests that their relative speed is lower there. We hypothesized that this can be caused by different G+C compositions of the introns versus exons in those genes. Supplementary Figure S10a shows that *S. cerevisiae* tRNA introns were 15% poorer in G+C content than exons. Thus it seems plausible that RNA pol III slows down in introns because the higher relative A+T content facilitates DNA unwinding and destabilizes the elongating bubble at the intron (68).

Finally, a detailed analysis of the nTR tRNA genes BioGRO signal showed that it correlated with both RNA pol III presence (as determined by ChIP; (31)) and the gene copy number, and also in the same way that the level of mature tRNAs in the cytoplasm did (Figure 6b–d). No significant differences in nTR were seen when comparing tRNAs with stronger or milder effects on growth when deleted (69): 10.46 ± 2.93 versus 10.20 ± 2.83. In conclusion, our data indicate that, in agreement with previous postulations (discussed in (31)), RNA pol III nTR seems constant per tRNA gene copy, regardless of the fact that encoded tRNAs decode preferred or rare codons (Supplementary Figure S10b). This confirms that this class of genes achieves its transcription level by increasing the copy number rather than by altering the TR (69).

## DISCUSSION

We have quantitatively mapped active RNA polymerases at a high resolution in the yeast *S. cerevisiae* following a new technique. This BioGRO method improves preceding techniques (18,19,22,32) in one of the following ways, or more: firstly, it avoids the use of radioactivity; secondly, it is strand-specific; thirdly, RNase A treatment increases mapping resolution; fourthly, biotin-UTP labelling permits a direct hybridization approach to avoid the introduction of potential mature RNA noise by sample amplification-related techniques. In principle, this method can be implemented to any eukaryotic organism for which tiling arrays exist. In addition to the work described here, we have successfully implemented BioGRO for *Candida albicans* and for cultured insect cells (Jordán-Pla A., Miguel A. and Pérez-Ortín J.E., unpublished).

Alternative genome-wide methods for high-resolution nRNA analyses based on run-on labelling and next-generation sequencing (NGS) have been published for animal cells (23,44) and for yeast (32). Whereas NGS has a huge potential thanks to its resolution and sensitivity, its drawbacks lie in possible contamination with mature RNAs, which are >300 times more abundant than nRNA in yeast (18,32), the potential artefacts caused by cDNA synthesis, and the most sequencing power is used to read RNA pol I and III transcripts. The problem of contamination with mature RNA is also feasible when following other mapping methods based on nRNA isolation from chromatin (18,19,70), which report purification of only 40 times. In BioGRO, however, presence of any residual mature RNA does not interfere as it is not biotinylated, thus it is not detectable with the Affymetrix proprietary biotin-labelling protocol. This problem becomes apparent in the discrepancies encountered when comparing our results with those of McKinlay *et al.* (32). In that paper, a strong nRNA signal peak is seen just after the TSS. The authors argue that it corresponds to a paused RNA pol, similarly to other eukaryotes (21). However, several independent studies have not evidenced that peak when they used nRNA mapping (18,19) or ChIP of RNA pol II (59; 71) techniques. Our results favour the idea that *S. cerevisiae* lacks paused RNA pol II at +50 bp, probably due to the different position of the +1 nucleosome (see (6,81)).

Several factors confirm that we detected bona fide active RNA polymerase profiles. The signal level in the BioGRO samples is similar between introns and exons (Supplementary Figure S4b) and extends beyond the TSS and pA sites (Figure 2c). This result is, in part, similar to that reported by Churchman and Weissman (18), who used NGS for fragmented nRNA. We detected 5′ extensions, which is in agreement with alternative methods (19) (Supplementary Figure S8), and an RNA polymerase activity signal beyond the polyadenylation site as expected when analysing bona fide nRNA.

The existence of a run-on signal before the mapped TSS (Figure 2) may indicate cryptic sense transcription in the promoter region (72,73). The extension of the nTR signal downstream pA site has already been pointed by Churchman and Weissman (18) as proof of nRNA identity. We used our data to map the average extension of RNA pol II post-pA elongation to an average of ∼150 bp (Figure 2c). This is much shorter than the distances observed in animals (reviewed in (60)), but is consistent with the highly compact organization of the yeast genome, which shows much shorter inter-ORF distances (74,75). The BioGRO profile at the 3′ end of the yeast genes completely differs from the previous RNA pol II profiles obtained by ChIP (6,59). Our data suggest that RNA pol II accumulates at 150 bp before the UA-rich (TATATA-like) site (64,76). This accumulation probably represents a region with the slowed elongation rate reported by other authors (77). In this region, no relative increase in backtracking occurs given that both BioGRO and ChIP profiles are coincident (Figure 5a). This accumulated RNA pol II forms elongation complexes (ECs) characterized by the presence of Ser2/Tyr1-phosphorylated CTD (65) and a set of elongation factors (Spt4–6, Elf1, Spn1), including the CPF complex, which cleaves pre-mRNA and then polyadenylates the new 3′ end (28,59). Cleavage + polyadenylation seems to help specific changes in RNA pol II because its total density diminishes (Figure 2a), but the density of the EC (and, therefore, the elongating/total ratio; Figure 5b) increases, which indicates local acceleration. This transient acceleration peaks a few nucleotides before the poly(A) site and coincides with a similar peak of Pab1 and Pub1 binding to nascent mRNA (28), and also with a peak obtained by the PAR-CLIP mapping of RNA pol II (78), which depends on the presence of the Ysh1 cleaving factor. This suggests that fully active RNA pol II favours pre-mRNA cleavage. As the BioGRO profile sharply drops after the pA site (Figure 5a), whereas total RNA pol II accumulates, we interpret that inactive molecules accumulate. This result coincides with the deceleration seen after the pA site in several model organisms (reviewed in (64)) and with the interpretation made by Schaughency *et al.* (78) published during the review process of this paper. In fact the peak of total RNA pol II after the pA site has been previously found by other authors (6,59) and has been seen to contain a different set of elongation factors (Nrb1, Nab3, Sen1, Pcf11) and ‘torpedo’ 5′-3′ exonuclease Rat1 (56), whereas mRNA is associated with the RNA15 factor (28). This RNA pol II has been recently shown to have decreasing Tyr1 phosphorylation (65). Thus the change in the CTD-phosphorylation code, the substitution of factors associated with either DNA or RNA or various interrelated aspects probably cause a functional change in RNA pol II, whose propensity to backtracking and/or releasing associated nRNA increases (Supplementary Figure S11). The inactive RNA polymerases detected in the termination region can hypothetically be run-on incompetent without backtracking by, for instance, the action of Rat1 after trimming nRNA. However, we have not found any *in vitro* evidence for such a consequence of Rat1 action on RNA pol II in the literature. On the contrary, elongating RNA pol II, treated *in vitro* with recombinant Rat1 complexes, retains its 3′ RNA end at the active site and its elongation capability (79). The results from Lemay *et al.* (80) also appeared during the review process of this paper in *Schizosaccharomyces pombe* and suggest the existence of backtracked RNA pol II after the poly(A) site, which can be released by nuclear exosome action (Supplementary Figure S11).

Both anti-termination and torpedo models (64,65) would be compatible with our results. Moreover, the minimum RNA pol II backtracking near the pA site coincides perfectly with the nucleosome-free region, which maps at the 3′ end of eukaryotic genes (Figure 5b). It has been interpreted that the presence of positioned nucleosomes after the pA site in mammals slows down speed (81). Thus backtracking in the termination region would be mediated by the influence of the nucleosome positioned downstream of the pA site and would favour transcription termination (81). The precise alternating pattern of BioGRO in the 5′ region, which is coincident in period terms (165 bp), but is the opposite in intensity terms to the known nucleosomal pattern (Figure 3a), strongly suggests a causal relationship between them. The BioGRO analysis of the *isw2* mutant, which exhibited a parallel shift of run-on peaks and nucleosomes, allowed us to confirm this causal relationship (Figure 3f). The coincidence found between the BioGRO/RNA pol II-ChIP peaks of RP genes and their internucleosomal valleys (Figure 4c), despite the shorter nucleosomal repeat of this gene category, is another piece of evidence that supports the negative influence of the positioned nucleosomes on RNA pol II activity. It has been shown both *in vitro* (82,83) and *in vivo* (1,13,62,84) that RNA pol II faces each nucleosome as a potential obstacle, which provokes a slowdown and/or backtracking. The NET-seq analysis in yeast uncovered peaks of stalling just before the nucleosome dyad for nucleosomes +2, +3 and +4, and just after the dyad in nucleosome +1; this scenario suggests that nucleosomes are the major source of RNA pol II pausing (18). Weber *et al.* (62) also found an accumulation of stalled RNA pol II before nucleosomes +1 and +2 in *Drosophila*. As stalling was better detected (yeast) or changed position (*Drosophila*) in the absence of TFIIS, it has been suggested to be caused by enhanced backtracking. Our results also evidence an antinucleosomal profile for RNA pol II-specific activity (Figure 4b). Interestingly, RNA pol II (not necessarily active) has been described to peak at nucleosomes +2, +3 and +4, placed about −40 bp from the dyad axes (18). Our analysis placed active RNA pol II peaks at about −60 from the dyad in nucleosomes +2, +3 and +4 (Figure 3a). Therefore, our results support a model in which RNA pol II, when encountering a nucleosome, slows down and its backtracking probability increases at around 40 bp before the dyad, when a H2A-H2B dimer is released to generate a free DNA loop (13,85,86). Then it increases speed to reach the maximum almost at the end of the nucleosome, which is when the second H2A-H2B dimer is placed (10,11) and the nucleosome flips backwards (87,88). In conclusion, our results support the *in vitro* models for RNA pol II transcription through a nucleosome (1,12).

The average profile of RNA pol II-specific activity (BioGRO/RNA pol II ChIP ratio) across the 5′ region showed the absolute maximum on nucleosome +1 (Figure 4b). This was not shared by RP genes, which lacked this prominent +1 peak and exhibited the absolute maximum of RNA pol II-specific activity in the +1/+2 internucleosomal region (Figure 4c). Nonetheless, TATA and TATA-like genes showed no major differences in this sense (not shown). Since RP genes are prone to RNA pol II backtracking (24,26), our observation suggests that nucleosome +1 might play a regulatory role in this respect. Recent results obtained in *Drosophila* have also indicated a singular role of nucleosome +1 in regulating RNA pol II elongation and have connected this singularity to histone variant H2AZ (62). This nucleosome is typically enriched in H2AZ (89). Our results reveal that the genes containing low levels of H2AZ in nucleosome +1 exhibit poorer RNA pol II-specific activity on it than the genes enriched in this histone variant (Figure 4e). The different H2AZ levels in these two gene sets are not due to distinct transcription levels because we chose a subset of highly transcribed genes for this comparison. Our results also match the differential BioGRO/RNA pol II ChIP profile of RP genes since it has been described that this gene category is particularly poor in H2AZ (59), a histone variant that favours elongation in yeast (63). Therefore, H2AZ appears to prevent RNA pol II from backtracking in yeast immediately after transcription initiation. This contrasts with the proposed role of H2AZ in *Drosophila* as a backtracking enhancer (62).

The comparison made between the high-resolution genome-wide profiles obtained after different RNA pol II mapping techniques offers a unique opportunity to discriminate between the transcription modes followed by different genes. For instance in Supplementary Figure S7, we show that the RP, TATA and TATA-like genes present different BioGRO and Rpb3-ChIP profile shapes. These differences not only imply that RP genes have a smaller proportion of active RNA pol II than the rest (Supplementary Figure S6d) but also confirm the diversity of different gene regulons in transcriptional elongation terms (24,51).

Finally, transcriptional run-on does not differentiate amongst nuclear RNA polymerases. Since it is known that RNA pol I represents ∼60% of total yeast transcription (90), we expected to obtain a very high signal on rDNA. In fact the BioGRO signal at the rDNA locus exceeded that of the average RNA pol II by over 50 times (data not shown). As there were ∼150 copies of the rRNA gene (91), the total nTR for the rDNA gene was >7500 times that of an average RNA pol II gene. This value is compatible with the expected ratio calculated from the global RNA pol II transcription data (26%; (92)) and after considering some 5000 RNA pol II-transcribed genes. However, RNA pol III transcribes ∼280 genes in *S. cerevisiae* and accounts for ∼14% of total transcription (92). Most of those genes (274) correspond to the tRNA type. Our results show that nTR was over 50 times higher for an average tRNA gene than for an average RNA pol II gene, which agrees with the expected value from total RNA pol III transcription. tRNA genes are very short, even shorter than nucleosome size (31), and belong to 42 isoacceptor families of 1–16 almost identical sequence tRNA members (69). For several organisms, it has been shown that the cytoplasmatic levels of various tRNA isoacceptors correlate positively with the tRNA family's gene copy number (93,94). Hence it is generally assumed that RNA pol III transcription is almost invariable per gene copy (see the Discussion in (69)). To date, and despite different mapped components of the RNA pol III machinery genome-wide existing in both yeast and humans ((31); reviewed in (95)), there is no study available on the transcription rate for tRNA genes in eukaryotes. There is only a reference for the nTR in yeast 5S gene, which has been calculated from electron microscopy studies to be ∼28 transcripts/min for each gene copy (96). Using the data discussed by Phizicky (29) of 3–6 million tRNAs/yeast cell cycle, we can infer for tRNA genes an average nTR per gene copy to be between 120 and 240 molecules/min, a figure that is ∼10 times higher than the TR for RNA pol I and 35 times that of the RNA pol II genes with the highest TR (histone genes; see (27)). As explained before, BioGRO is able to detect nTR for all tRNA genes with high sensitivity. We found similar correlations for nTR, the amount of tRNA for each isoacceptor family and the gene copy number (Figure 6b–d). Therefore, our results confirm the hypothesis that RNA pol III is poorly regulated at the individual gene level and that the main purpose (but perhaps not the only one) of the multiplicity of tRNA gene copies is to gradate the mature tRNA species level in the cytoplasm.

In summary, high-resolution run-on mapping reveals significant quantitative variations in RNA pol II and III activity along their genes in relation to initiation and termination phases, and reveals, for the first time *in vivo*, a strong dependency of RNA pol II elongation activity on nucleosome positioning. Such nucleosome dependence causes gene-specific profiles and reveals that RNA pol II-dependent genes differ not only at the transcription initiation level, as generally acknowledged (97), but also at the elongation level. This novel perspective involves inactivation/reactivation as an important aspect of RNA polymerase dynamics throughout the transcription cycle.

## ACCESSION NUMBER

The GEO accession number for new the BioGRO and RNA pol II ChIP data is GSE58859.

## SUPPLEMENTARY DATA

Supplementary Data are available at NAR Online.

SUPPLEMENTARY DATA
