# Supplementary material for: Chromatin-dependent regulation of RNA polymerases II and III activity throughout the transcription cycle
Source: Nucleic Acids Res. 2014 Dec 29;43(2):787–802. doi: 10.1093/nar/gku1349 (PMC4333398; doi:10.1093/nar/gku1349)
Supplement: SUPPLEMENTARY DATA [file supp_43_2_787__index.html]

Chromatin-dependent regulation of RNA polymerases II and III activity throughout the transcription cycle — SUPPLEMENTARY DATA 

# Chromatin-dependent regulation of RNA polymerases II and III activity throughout the transcription cycle

## SUPPLEMENTARY DATA

**Files in this Data Supplement:**

- SUPPLEMENTARY DATA
