# Supplementary material for: Chromatin-dependent regulation of RNA polymerases II and III activity throughout the transcription cycle
Source: Nucleic Acids Res. 2014 Dec 29;43(2):787–802. doi: 10.1093/nar/gku1349 (PMC4333398; doi:10.1093/nar/gku1349)
Supplement: SUPPLEMENTARY DATA [file supp_gku1349_nar-02425-m-2014-File003.pdf]

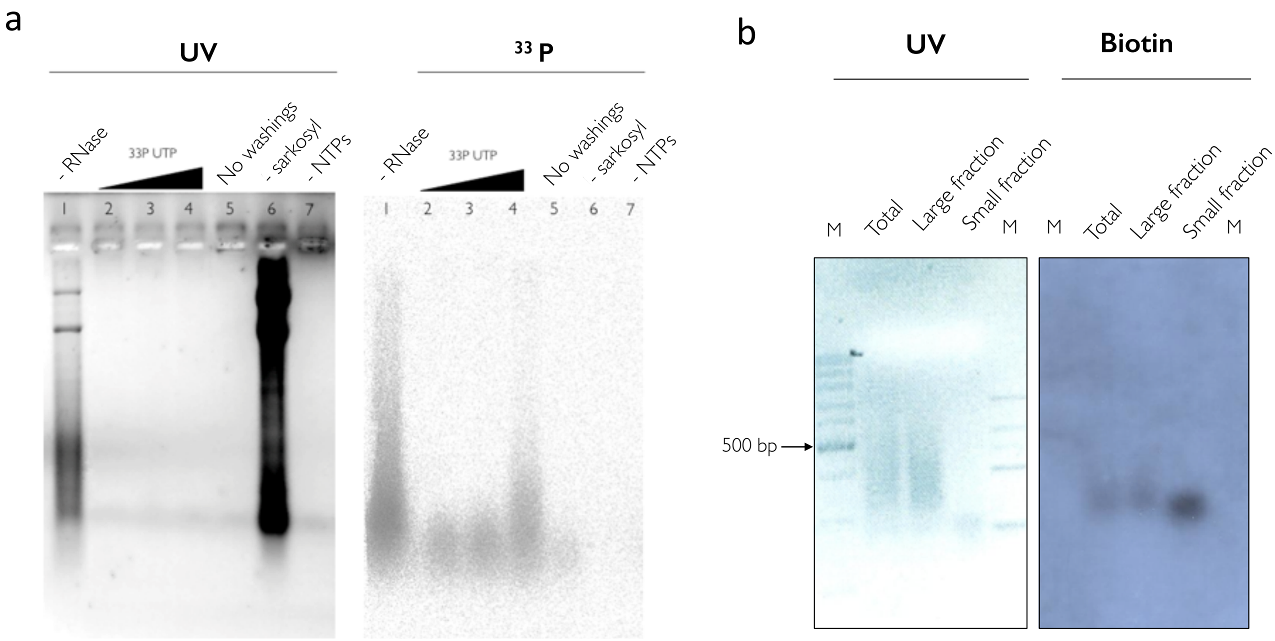

Figure S1. Electrophoretic analyses of the BioGRO method. (a) Incorporation of radioactive nucleotides by run-on under different conditions. The figure shows the same denaturing agarose gel stained with EtBr (left), developed for radioactivity detection (right). Sample 1 corresponds to a run-on in which degradation with the RNase step has been skipped. Samples 2-4 are the run-ons digested with RNase, marked in the presence of increasing concentrations of radioactive nucleotide (3 µM, 6 µM, 12 µM). Sample 5 is a run-on digested with RNase, which was not eliminated later. Sample 6 corresponds to a negative control with no previously permeabilized cells, and sample 7 is the negative control carried out in the reaction in the absence of nucleotides. (b) Nascent RNA fractionation after the run-on using Biotin-UTP instead of ^{33}P-UTP and the columns as described in M&M. The figure shows the same native agarose gel stained with EtBr (left), and then developed with anti-Biotin (right). All the sample lanes in both figures were loaded with the same amount of RNA, except for lane 6 in Fig. S1A (10X more).

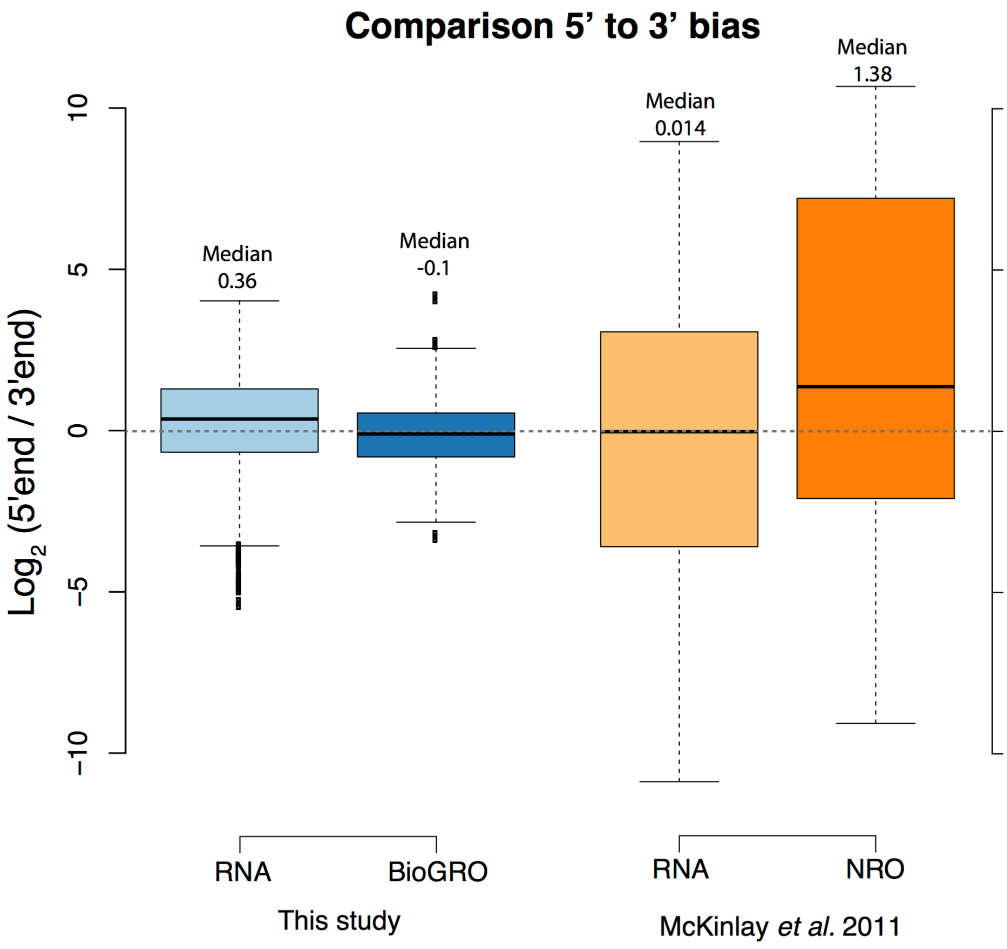

Figure S2. Comparison of the BioGRO method results and those of McKinlay et al. A boxplot comparison between the signal of the 5' and the 3' side of each gene measured by either tiling array in blue (Total RNA and BioGRO, in this study) or high-throughput sequencing in orange (Total RNA or Nuclear run-on from McKinlay *et al*, ref 32). We compared the signal of their most 5' region (500 bp) with their most 3' region (500 bp). Boxes represent the median (central line) and the first and third quartiles. Only the confident genes longer than 1 Kb (BioGRO signal >6 times over the background) were used for the analysis. Only the run-on sample from McKinlay *et al* has a big 5'/3' bias. This could be due to the absence of RNase treatment prior to purifying nascent RNAs.

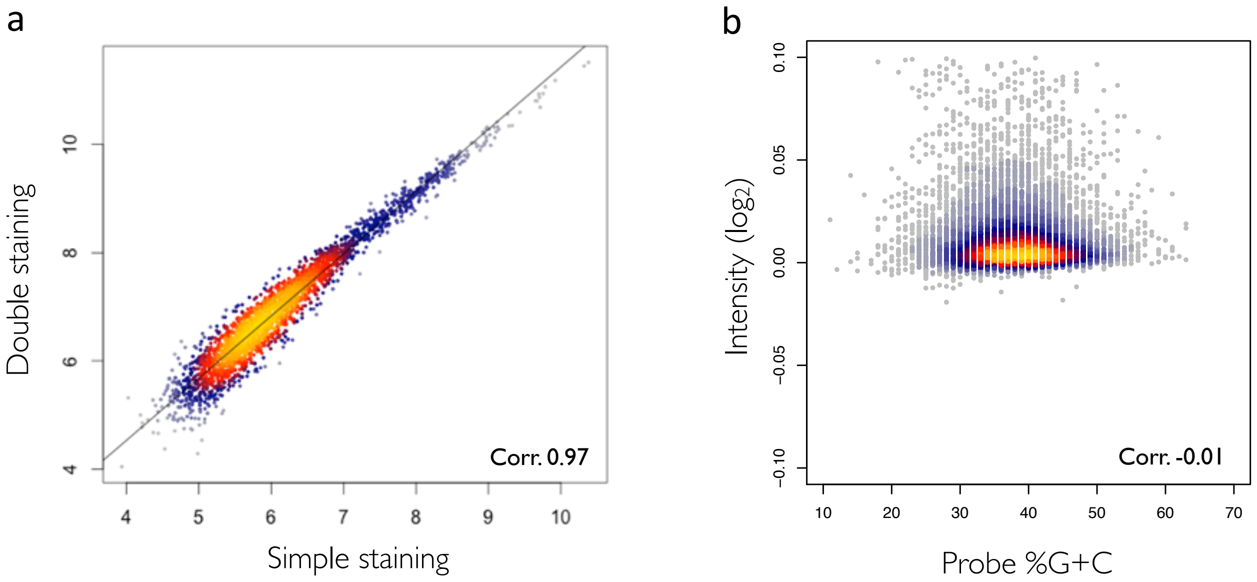

Figure S3. Analyses of the potential biases of the BioGRO signals. (a) Double microarray staining increases the signals of all the probes without biasing the results. (b) There is no bias in probe intensity in relation to G+C content. This may be due to either the low incorporation efficiency of Biotin-UTP, which provokes similar labeling for all fragments, and/or the compensatory effect of the poor hybridization efficiency of U-rich fragments.

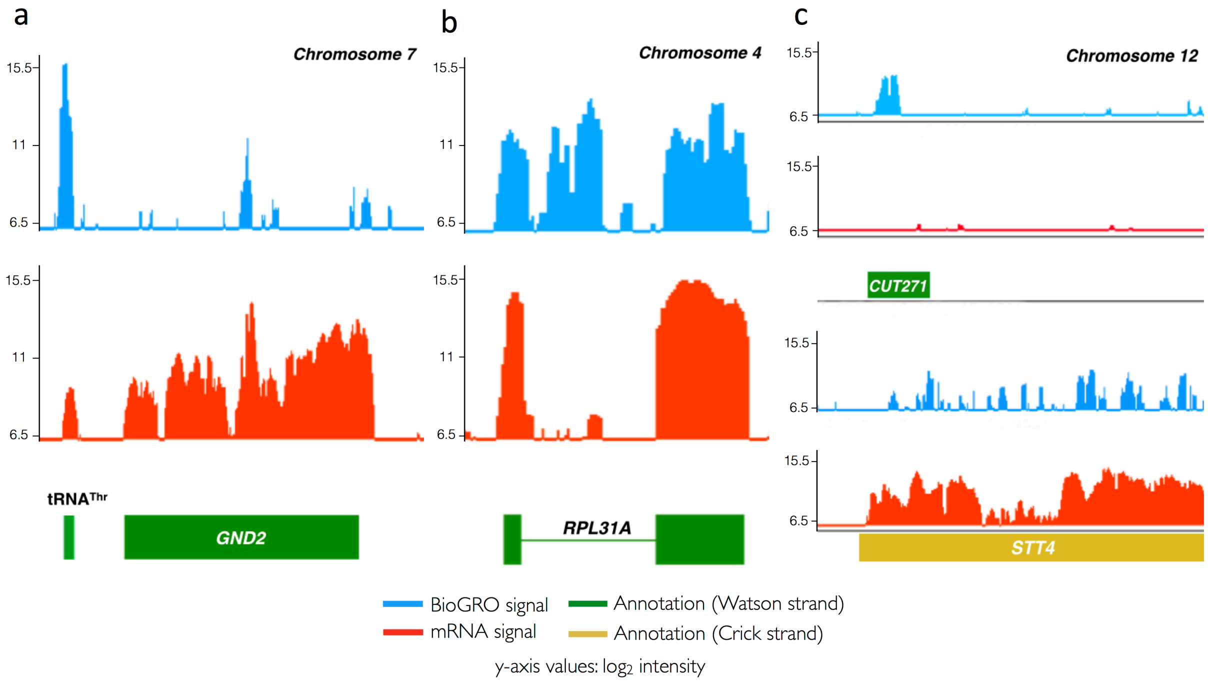

Figure S4. Some examples of the BioGRO signal along individual genome regions. (a) Example of a tRNA gene showing a very intense peak in BioGRO (blue) if compared with the same region in a conventional cDNA analysis (orange). (b) Introns show a BioGRO signal that is not seen in the mature mRNA signal. Example of the *RPL31A gene region*. (c) BioGRO detects some AS transcripts, such as the *CUT271* transcript (37). Intensity measurements (Y-axes) are taken on a $\log_2$ scale.

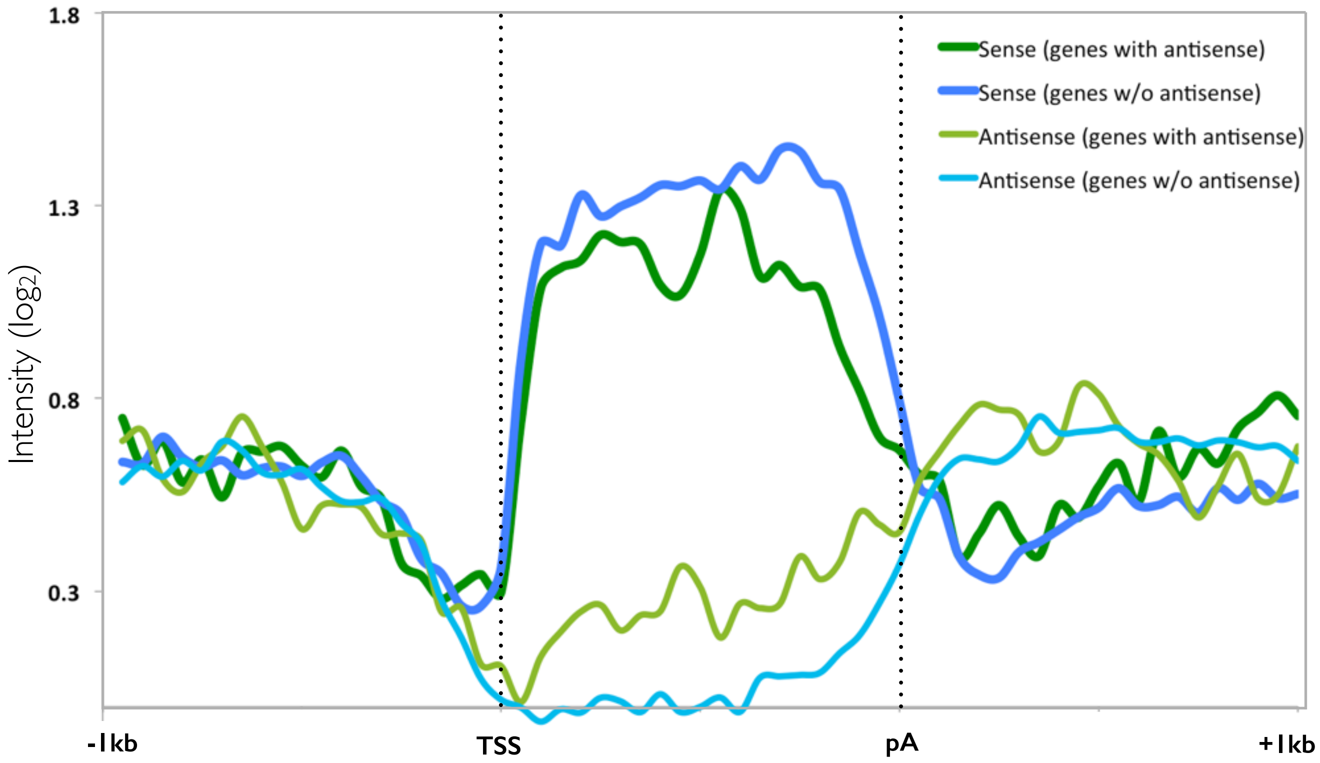

Figure S5. The metagene BioGRO profile for the genes with or without antisense transcription. Distance is on a real scale before the TSS and after pA and in normalized units for all the gene lengths in the transcribed region. The plateau along the transcribed region is higher in the genes with no AS transcription, whereas the intensity in the antisense strand is lower in the genes with no AS transcription, as expected.

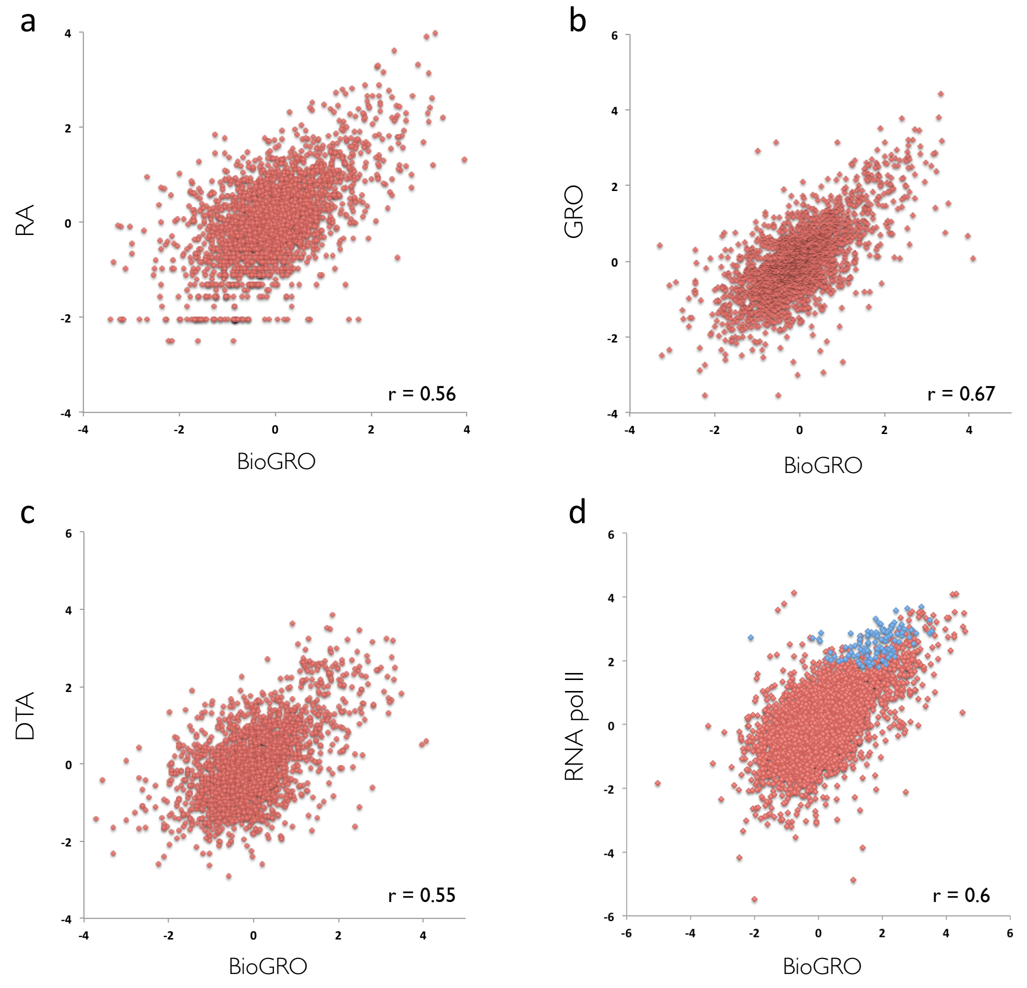

Figure S6. Comparisons of the BioGRO results with other genome-wide transcription-related results. Comparisons of signals for: (a) mRNA levels (from ref. 48); (b) GRO (from 27); (c) dynamic transcriptome analysis (DTA, from 49); and (d) RNA pol II ChIP (anti-Rpb3, this paper). RP genes are marked as blue dots in (d). r indicates Pearson's correlation coefficient.

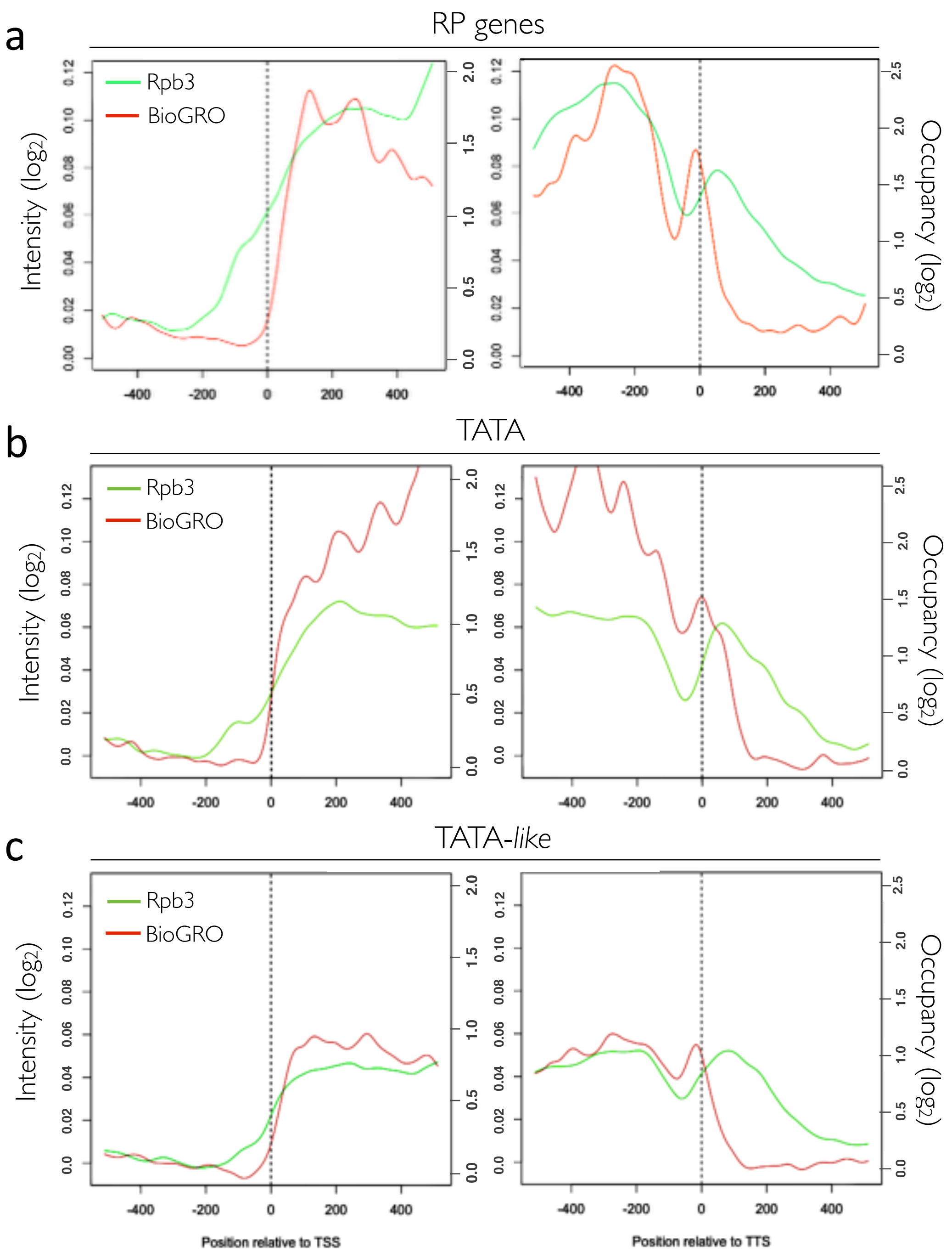

Figure S7. Average profiles for the 5' and 3' regions in TATA, TATA-like and RP genes. (a) RP genes show characteristic profiles at both gene ends. Average profiles for BioGRO (red lines) and ChIP-Rpb3 (green lines) in both the 5' and 3' regions in TATA (b) and TATA-like (c). Only the genes that were highly expressed for both TATA and TATA-like from the 809 gene set are computed in this graph instead of averaging all the genes, as in Fig. 3B.

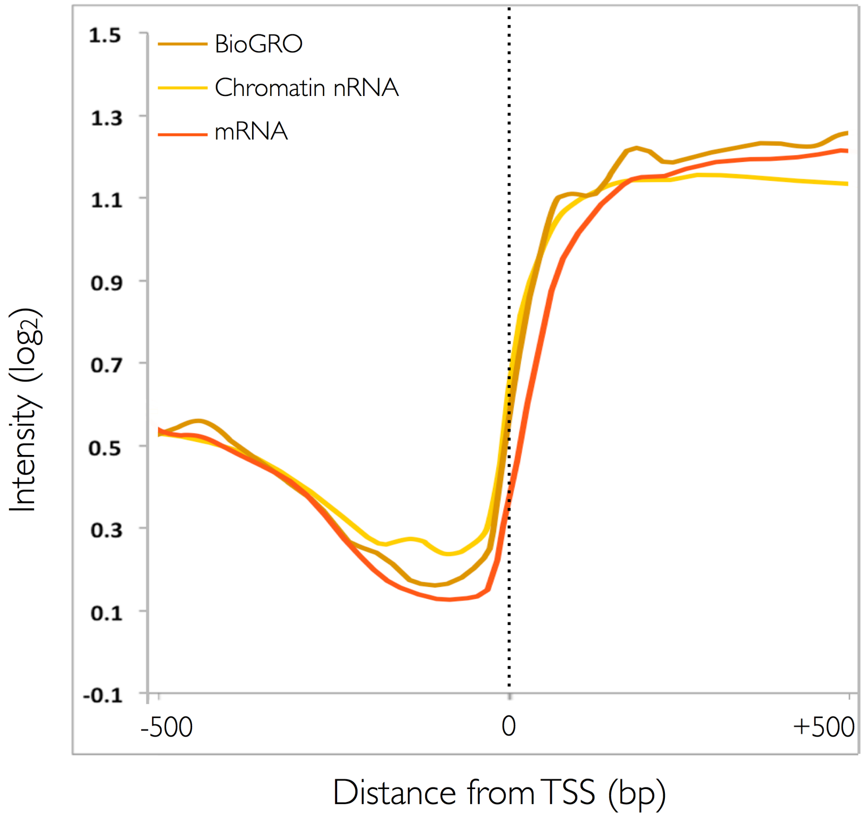

Figure S8. Comparison of the metagene profiles in the 5' region for the RNA pol II genes in BioGRO and chromatin-bound nascent RNA. The BioGRO data from this study were compared with chromatin-associated nRNA obtained by Carrillo-Oesterreicher et al., (19) and with the mRNA profile from ref. (37). As seen, both nRNA mapping methods gave profiles that advanced about 70 bp to the mRNA profile.

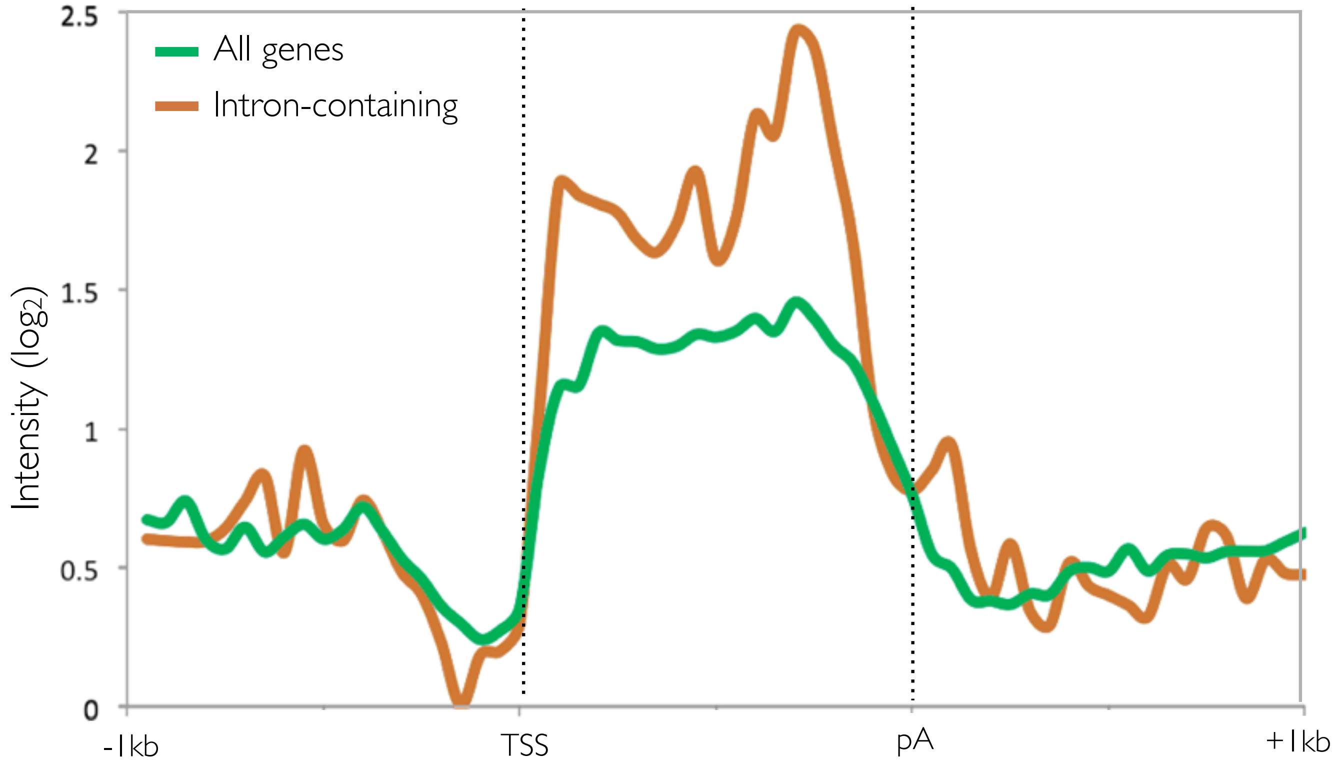

Figure S9. The metagene BioGRO profile for the genes with or without introns. A similar plot to that shown in Figure S5, but by separating the genes with an intron (orange) vs. the remaining genes (green). Enrichment in the 3’ region, if compared to the 5’ region, is seen in the genes with introns, whereas the general profile is flat.

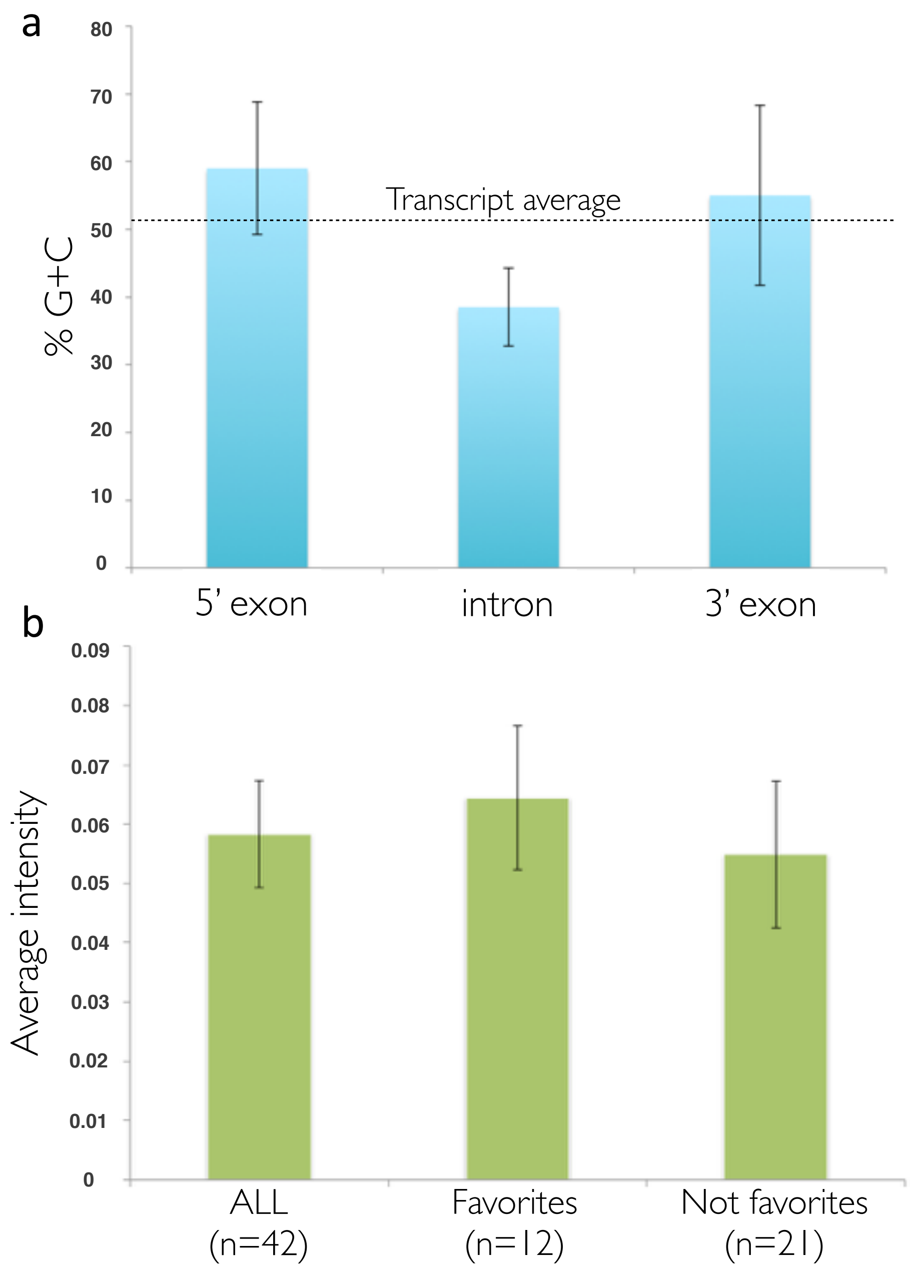

Figure S10. tRNA genes in *S. cerevisiae*. (a) Comparisons of the average C+G content in tRNA genes. A comparison between exons and introns. Introns have a significantly smaller G+C content than exons. (b) Comparison of the average BioGRO intensities in favorite and non-favorite tRNAs genes. There is no significant difference in nTR between both gene kinds.

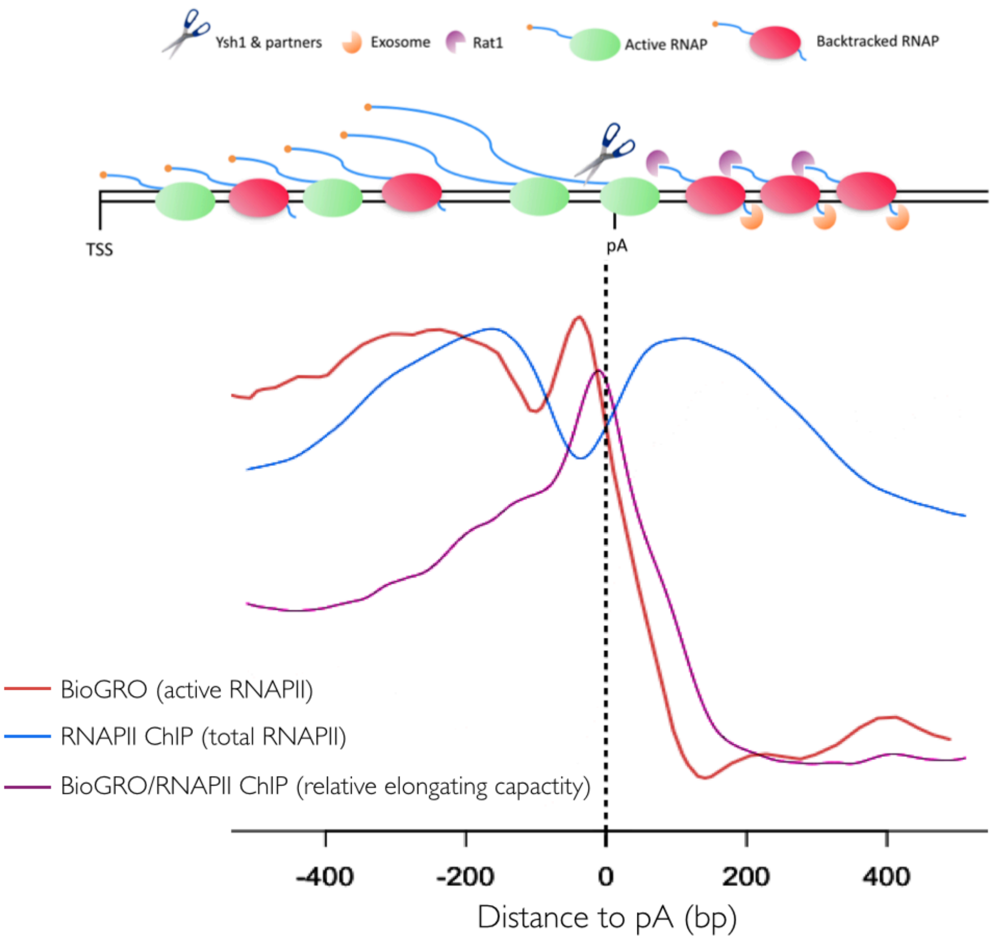

Figure S11.- Interpretation of ChIP, BioGRO and BioGRO/ChIP profiles. We interpret that ChIP profiles represent the total amount of RNA pol II. If one considers a small or negligible drop-off (see Sun et al., 2013), the amount of RNA pol and the elongation rate will be inversely related (see Ehrensberger et al., 2013). The BioGRO signal, however, only takes the active RNA pol II and is related not only to speed. The BioGRO/ChIP ratio represents the proportion of active polymerases. In fact as the intensity of the signal for both ChIP and BioGRO is obtained from a population of cells, it represents the average of those that have either RNA pol II or active RNA pol II. Therefore when increasing the BioGRO/ChIP ratio, the meaning is that a larger proportion of polymerase molecules is elongating, thus the average speed for the cell population at that precise genome site will be faster. This does not mean that the RNA pol molecule displays faster enzymatic velocity, but has a smaller number of stops. Following this argument, it can be concluded that RNA pol II accelerates at the pA site. The particular behaviour of RNA pol molecules is not seen in this analysis. Therefore, it is still possible that individual molecules pause or decelerate. In any case, pausing or deceleration does not necessarily involve backtracking, which reflects a totally different situation for RNA pol II. The absence of nucleosomes around the poly(A) site is coherent with backtracking being absent. In a recent paper, Lemay et al. (80) propose that the nuclear exosome competes with mRNA processing and helps.

Additional References not cited in the paper.

Ehrensberger AH, Kelly GP, Svejstrup JQ. 2013. Mechanistic Interpretation of Promoter-Proximal Peaks and RNAPII Density Maps. Cell 154(4):713–715.

Sun M, Schwalb B, Pirkl N, Maier KC, Schenk A, Failmezger H, Tresch A, Cramer P. 2013. Global analysis of eukaryotic mRNA degradation reveals xrn1-dependent buffering of transcript levels. Mol Cell 52(1):52–62.
